# Assessment of Luminal and Basal Phenotypes in Bladder Cancer

**DOI:** 10.1038/s41598-020-66747-7

**Published:** 2020-06-16

**Authors:** Charles C. Guo, Jolanta Bondaruk, Hui Yao, Ziqiao Wang, Li Zhang, Sangkyou Lee, June-Goo Lee, David Cogdell, Miao Zhang, Guoliang Yang, Vipulkumar Dadhania, Woonyoung Choi, Peng Wei, Jianjun Gao, Dan Theodorescu, Christopher Logothetis, Colin Dinney, Marek Kimmel, John N. Weinstein, David J. McConkey, Bogdan Czerniak

**Affiliations:** 1 Department of Pathology, The University of Texas MD Anderson Cancer Center, Houston, TX USA; 2 Department of Bioinformatics and Computational Biology, The University of Texas MD Anderson Cancer Center, Houston, TX USA; 3 Department of Biostatistics, The University of Texas MD Anderson Cancer Center, Houston, TX USA; 4 Department of Environmental Health, University of Cincinnati, Cincinnati, Ohio USA; 5 Johns Hopkins Greenberg Bladder Cancer Institute, Johns Hopkins University, Baltimore, MD USA; 6 Department of Genitourinary Medical Oncology, The University of Texas MD Anderson Cancer Center, Houston, TX USA; 7 Samuel Oschin Comprehensive Cancer Institute, Cedars-Sinai, Los Angeles, CA USA; 8 Department of Urology, The University of Texas MD Anderson Cancer Center, Houston, TX USA; 9 Department of Statistics, Rice University, Houston, TX USA

**Keywords:** Cancer, Oncology, Urology

## Abstract

Genomic profiling studies have demonstrated that bladder cancer can be divided into two molecular subtypes referred to as luminal and basal with distinct clinical behaviors and sensitivities to frontline chemotherapy. We analyzed the mRNA expressions of signature luminal and basal genes in bladder cancer tumor samples from publicly available and MD Anderson Cancer Center cohorts. We developed a quantitative classifier referred to as basal to luminal transition (BLT) score which identified the molecular subtypes of bladder cancer with 80–94% sensitivity and 83–93% specificity. In order to facilitate molecular subtyping of bladder cancer in primary care centers, we analyzed the protein expressions of signature luminal (GATA3) and basal (KRT5/6) markers by immunohistochemistry, which identified molecular subtypes in over 80% of the cases. In conclusion, we provide a tool for assessment of molecular subtypes of bladder cancer in routine clinical practice.

## Introduction

Bladder cancer develops in the stratified epithelial layer of the urinary system along two tracks referred to as papillary and nonpapillary^1–4^. These two forms of the disease have distinct but somewhat overlapping molecular profiles and are approached clinically by different management plans^5–9^. Recent genomic investigations revealed that bladder cancer is characterized by complex molecular alterations, heavy mutational load, and frequent involvement of a unique subset of chromatin remodeling genes^10–12^. They have shown that expression signatures can be linked to cancer progression, metastases, and survival^9,13–16^. These studies also provided the evidence that molecular diversity of bladder cancer underlies a spectrum of its clinical behavior and responses to therapy^15–21^.

Several groups of investigators used genome expression profiling to classify bladder cancer into various molecular subtypes which showed remarkable similarity to major intrinsic subtypes originally identified in human breast cancers and referred to as luminal and basal^10,11,22–27^. The markers characteristic of these two major groups reflect the expression signature of normal basal and intermediate/luminal urothelial cell layers. Most importantly these two subtypes show distinct clinical behaviors and responses to chemotherapy^18,22,28–30^. In a chemotherapy naive setting, basal cancers are more aggressive than luminal subtypes, but they are associated with more survival benefit from frontline platinium-based therapy.

Here we reanalyzed the data from The Cancer Genome Atlas (TCGA) and several MD Anderson Cancer Center (MDACC) bladder cancer sample sets with a focus on their molecular subtypes. We aimed to develop a quantitative algorithm for the assessment of luminal and basal phenotypes in bladder cancer and validated a simple immunohistochemical classifier which can be used in routine pathology practice to identify the intrinsic molecular subtypes of bladder cancer in primary care centers.

## Results

**Figure 1:**
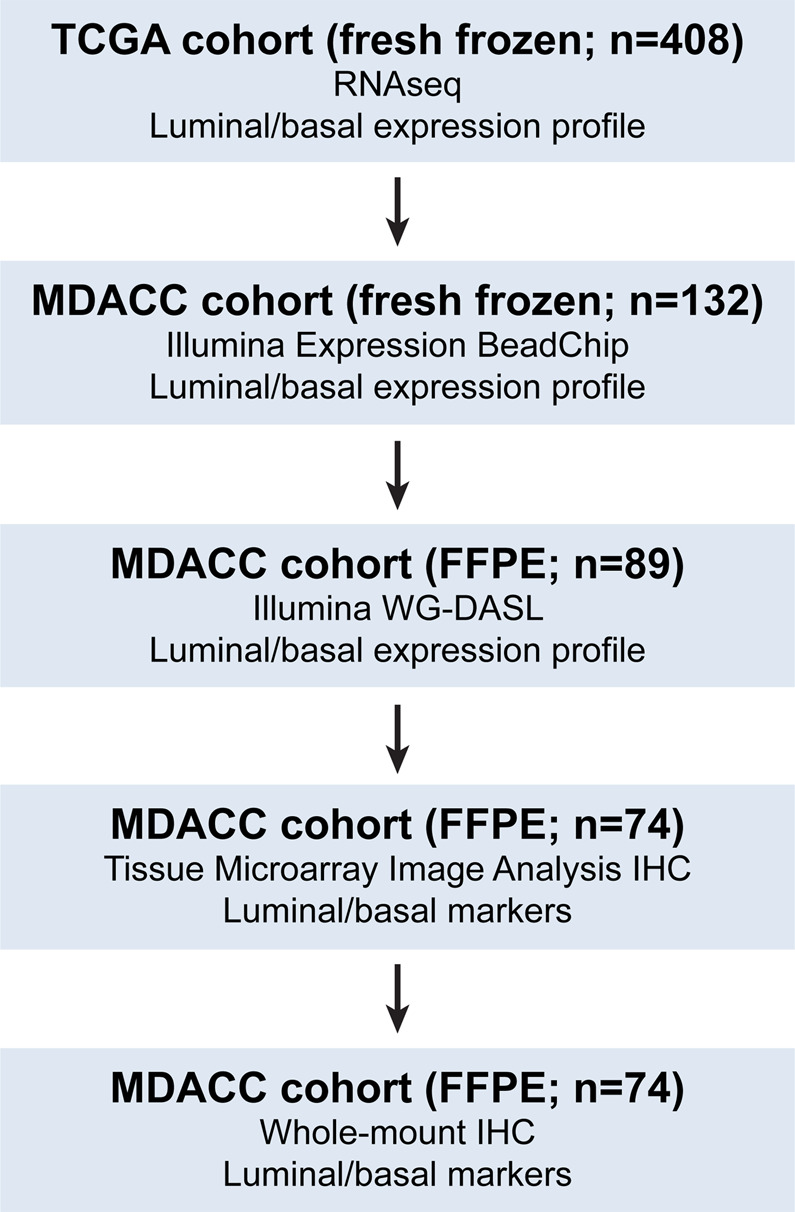
Organizational flow-chart of analyses on different cohorts of bladder cancer samples. The TCGA cohort (n = 408) was used as a training set to develop the BLT scores. None of the cases of the MDACC fresh frozen cohort (n = 132) and MDACC FFPE cohort (n = 89) used as validation for the BLT score overlapped with the TCGA. A part of the MDACC FFPE cohort (n = 74) was used to construct the tissue microarray in which the expression of selected luminal and basal markers was validated by quantitative image analysis. The MDACC cohort of whole-mount sections (n = 74) corresponded to the MDACC fresh frozen cohort was used for semi-quantitative assessment of immunohistochemical classifier of molecular subtypes.

**Figure 2:**
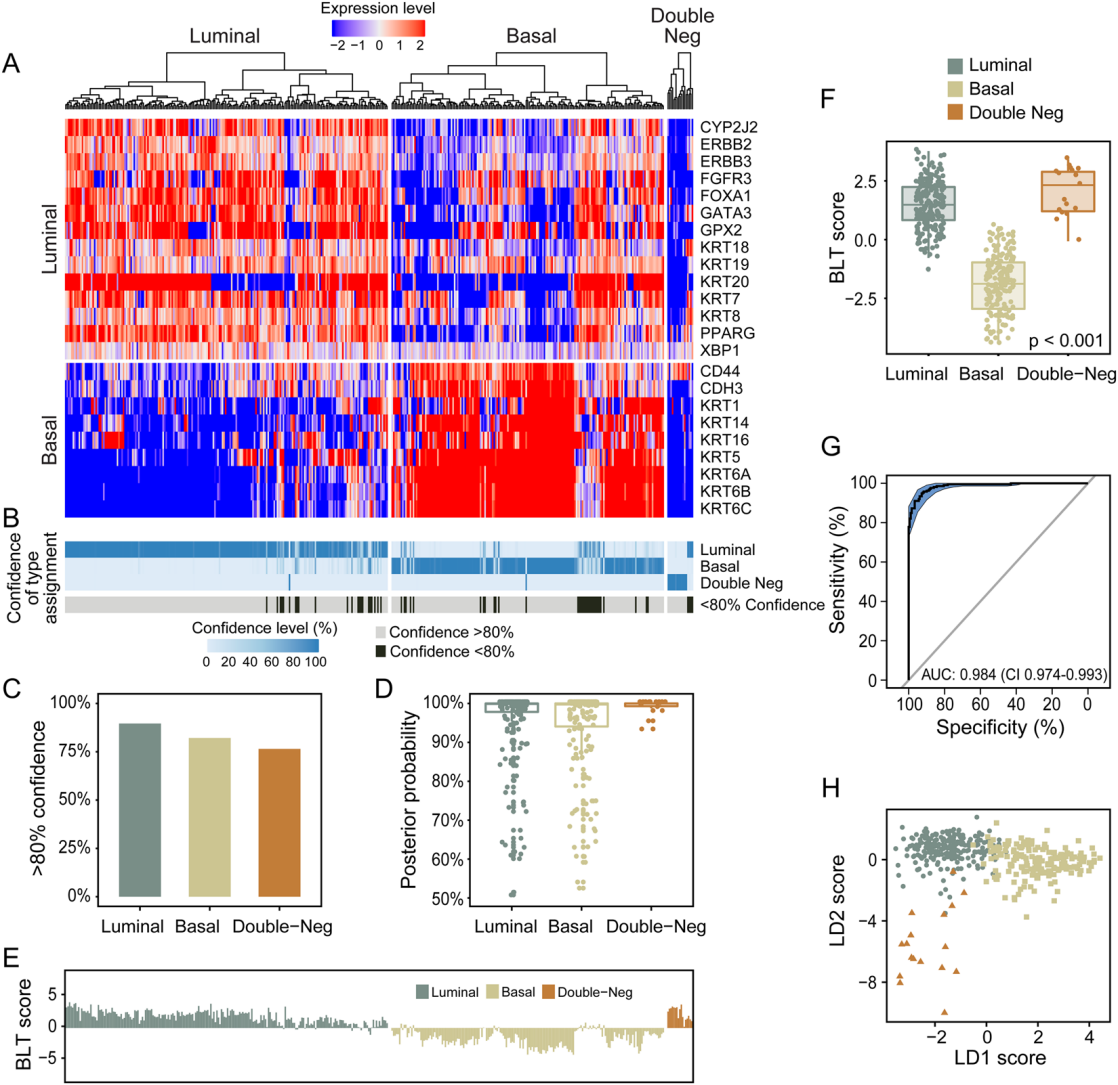
Whole-transcriptome mRNA expression profiling of the TCGA bladder cancer cohort (n = 408). (**A**) Hierarchical clustering with luminal and basal markers. (**B**) Prediction strengths of molecular subtypes. (**C**) Proportion of cases with >80% prediction strength of the molecular subtypes. (**D**) Box plot analysis of prediction strengths for individual cases by posterior probability. (**E**) Basal to luminal transition (BLT) scores in molecular subtypes of bladder cancer. (**F**) Box plot analysis of BLT scores in molecular subtypes of bladder cancer. (**G**) ROC curve of BLT scores segregating luminal and basal subtypes of bladder cancer. (**H**) Scatter plot of two-dimensional linear discriminant LD1 and LD2 scores in luminal, basal, and double-negative subtypes of bladder cancer. The panels in A and B were generated using the R package ComplexHeatmap (version 1.14.0). Panels C, D, E, F, and H were generated using the R package ggplot2 (version 3.2.1). Panel G was generated with pRoc (version 1.8).

The plan of the study to analyze luminal and basal phenotypes in several bladder cancer cohorts is outlined in Fig. 1. To evaluate the molecular subtypes of bladder cancer we initially analyzed the mRNA expression in the TCGA cohort (n = 408 cases) of invasive bladder cancers^11^. Our hierarchical clustering using signature luminal and basal markers revealed two major subtypes referred to as luminal and basal as well as a small subset of samples which were negative for both groups of markers. **(**Fig. 2A**)** The first cluster referred to as luminal (n = 212 cases) was characterized by the expression of markers such as KRT20, GATA3, FOXA1, XBP1, and CD24 which were associated with terminal urothelial differentiation. The second cluster (n = 179 cases) referred to as basal displayed strong expressions of basal genes such as high molecular weight keratins (KRT5, KRT6, and KRT14), CDH3, and CD44. In addition, a small subset of double-negative tumors (n = 17 cases) did not show expression of either luminal and basal genes. The algorithm of cluster prediction strengths designed by R. Tibshirani *et al*. showed that the prediction of luminal versus basal subtypes was 95.9%^31^. The prediction strength of double-negative subtype versus basal or luminal was 67.7% and 71.3% respectively. The analyses of the prediction strengths of individual cases in their respective molecular subtypes by posterior probability as defined by Bayes theorem showed that the majority of cases had their class assignment with strength >80%. **(**Fig. 2B–D**)**^32^. The basal cluster was heterogeneous and contained a subset of samples which coexpressed basal and luminal markers. The majority of cases from this subgroup showed, however strong (>80%) assignment to the basal subtype. In order to quantitatively assess the basal and luminal phenotypes in bladder cancer we developed the BLT score using the expression levels of 28 luminal and 20 basal marker genes **(**Supplementary Table 4**)**. We used a LDA model to assess their power to isolate the three molecular subtypes i.e. luminal, basal, and double negative. LASSO was used to select the best 16 and 12 luminal and basal marker genes and develop a quantitative measure of the BLT score. **(**Supplementary Table 4**)** Cancer of luminal type in the vast majority of cases (98%) had positive BLT scores. In contrast, basal cancers showed negative values in 85% of the cases. **(**Fig. 2E,F**)** By analyzing the ROC curve for the BLT scores (AUC 0.984; 95% confidence interval [CI] = 0.974–0.993) we identified the optimal cutoff point which segregated the luminal and basal subtypes with 93.9% sensitivity and 92.5% specificity. **(**Fig. 2G**)** Then the LDA model was applied using the expression of marker genes selected by LASSO and the resulted LD1 and LD2 scores represented the top two combinations of gene expression that maximized the separation of not only luminal and basal subtypes but also double-negative. **(**Fig. 2H**)** The five fold cross validation model showed that LD1 and LD2 scores identified the three subtypes with 91.9% accuracy.

**Figure 3:**
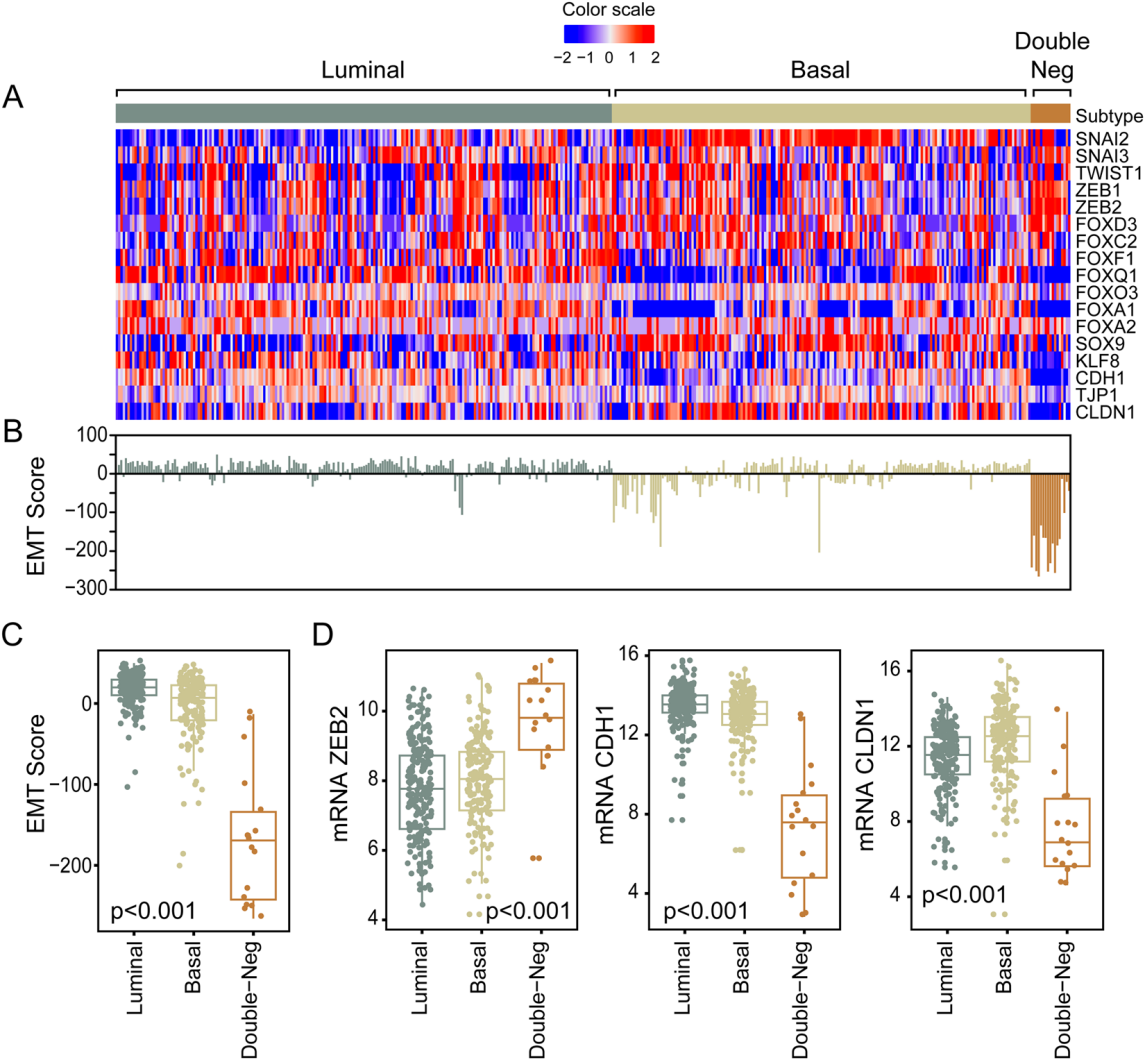
Dysregulation of the EMT network in the TCGA bladder cancer cohort (n = 408). (**A**) Expression pattern of representative genes in the EMT regulatory network. (**B**) EMT scores in molecular subtypes of bladder cancer. (**C**) Box plot of EMT scores in molecular subtypes of bladder cancer. (**D**) Box plot analyses of expression levels of a signature transcription factor (ZEB2) and adhesion molecules (CDH1 and CLDN1) involved in EMT. Panel A was generated using the R package ComplexHeatmap (version 1.14.0). Panels B–D were generated using the R package ggplot2 (version 3.2.1).

In order to better characterize the molecular subtypes of bladder cancer we performed additional analyses of their EMT and immune profile status. To assess the status of EMT we analyzed the expression signature of transcriptional factors involved in the activation of EMT. **(**Fig. 3A**)** They included the members of the SNAIL, TWIST, ZEB, FOX, SOX, and KLF families. These analyses were complemented by the assessment of the expression levels of homotypic adhesion molecules such as E-cadherin (CDH1), claudin 1 (CLDN1), and tight junction protein 1 (TJP1) characteristic of epithelial phenotype. The downregulation of the expression levels of these genes is a signature feature of the active EMT state. The luminal and basal subtypes of bladder cancer were characterized by positive EMT scores consistent with their epithelial phenotype. **(**Fig. 3B**)** The tumors of the double-negative category had significantly lower EMT scores reflecting their activated EMT state. **(**Fig. 3C**)** Accordingly several members of the transcription factors involved in activations of EMT were upregulated in double-negative tumors, which also showed the downregulation of signature adhesion molecules such as CDH1 and CLDN1 **(**Fig. 3D).

**Figure 4:**
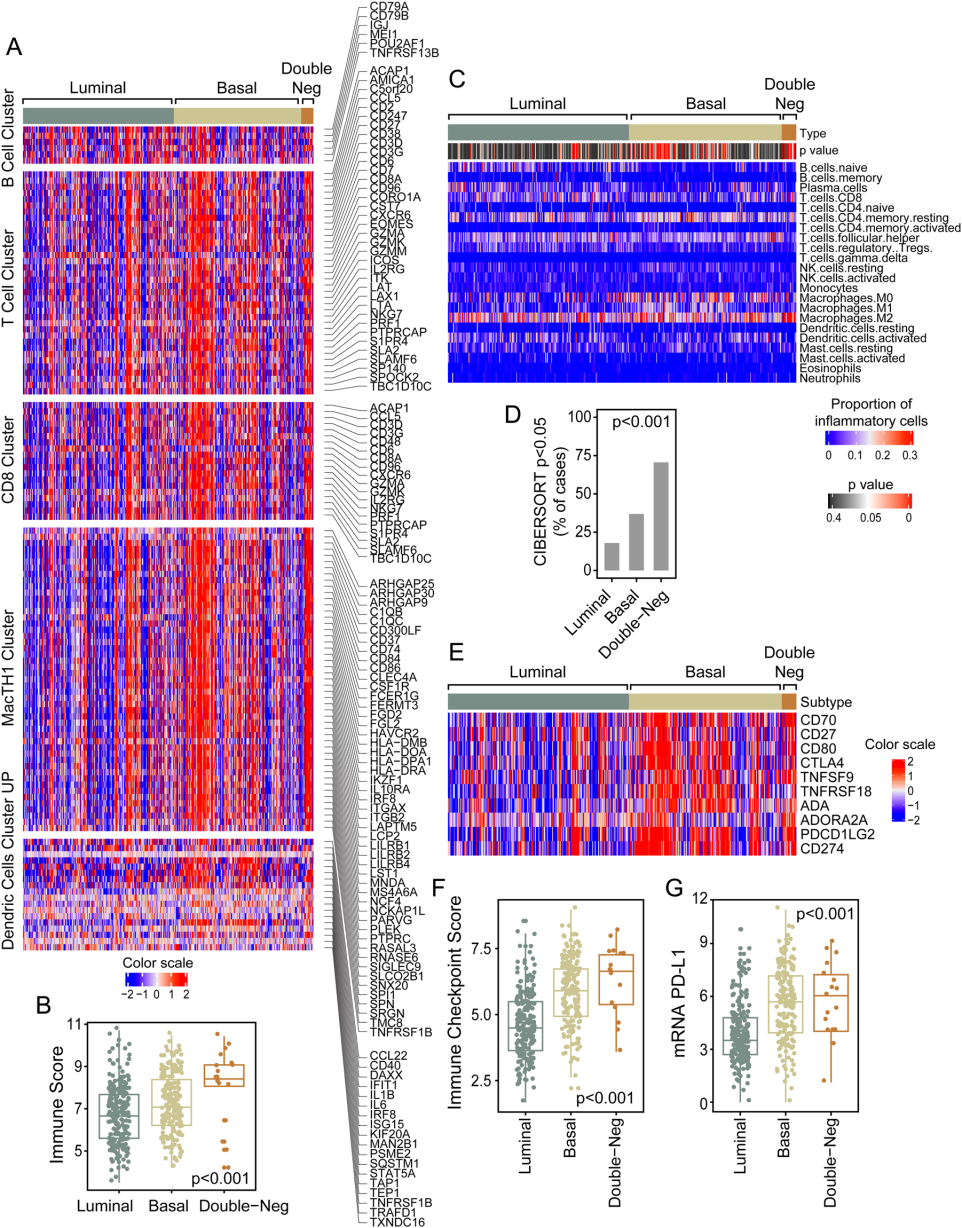
Immune signature in the TCGA bladder cancer cohort (n = 408). (**A**) Expression pattern of immune cell infiltrate in molecular subtypes of bladder cancer. Top to bottom: B cell, T cell, CD8, MacTH1, and dentritic cell expression clusters. Boxed areas identify samples with enrichment of immune cell infiltrate. (**B**) Box plot of immune scores calculated using the expression profile shown in A in molecular subtypes of bladder cancer. (**C**) Heatmap of CIBERSORT scores for 22 immune cell types in molecular subtypes of bladder cancer. (**D**) Proportion of cases with significant CIBERSORT score in molecular subtypes of bladder cancer. (**E**) Expression of immune checkpoint genes in molecular subtypes of bladder cancer. (**F**) Box plot of immune checkpoint scores calculated using the gene expression profile in (**E**). (**G**) Box plot of mRNA PD-L1 expression levels in molecular subtypes of bladder cancer. Panels A, C, and E were generated using the R package ComplexHeatmap (version 1.14.0). Panels B, D, F, and G were generated using the R package ggplot2 (version 3.2.1).

Since immune checkpoint blockade is clinically active in approximately 15% of patients with bladder cancer and this response is associated with infiltration of the tumor by activated cytotoxic lymphocytes as well as with specific molecular subtypes, we analyzed the status of immune related genes. **(**Fig. 4A**)** Basal and double-negative tumors were characterized by increased immune signature. **(**Fig. 4B**)** More in depth analyses of immune infiltrate was performed using CIBERSORT algorithm which provided quantitative assessment of expression signature of 22 immune cell types. **(**Fig. 4C**)** It confirmed the initial observation and showed that basal and double-negative molecular subtypes had progressively increasing immune infiltrate as compared to luminal tumors. **(**Fig. 4D**)** Similar to the immune infiltration, the analyses of the expression signature of immune checkpoint ligands and their receptors showed that virtually all of them were overexpressed in basal and double-negative tumors. **(**Fig. 4E,F**)** This included the overexpression of the key therapeutic target gene of immune checkpoint blockade (PD-L1) overexpressed in basal and double-negative tumors as compared to luminal subtyp **(**Fig. 4G).

**Figure 5:**
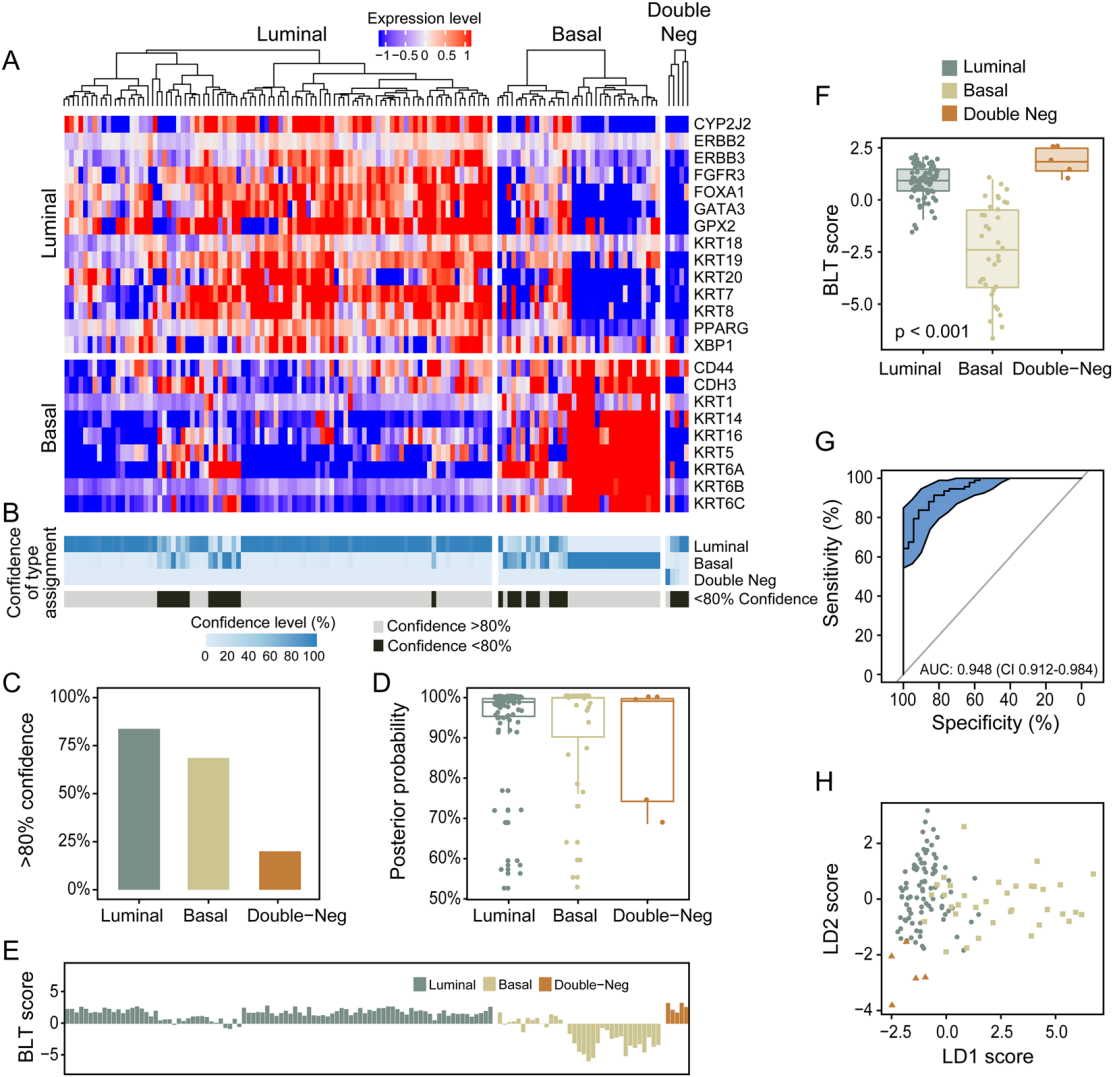
Whole-trancriptome mRNA expression profiling of the MDACC fresh frozen bladder cancer cohort (n = 132). (**A**) Hierarchical clustering with luminal and basal markers. (**B**) Prediction strengths of molecular subtypes. (**C**) Proportion of cases with >80% prediction strength of the molecular subtypes. (**D**) Box plot analysis of prediction strength for individual cases by posterior probability. (**E**) BLT scores in molecular subtypes of bladder cancer. (**F**) Box plot analysis of BLT scores in molecular subtypes of bladder cancer. (**G**) ROC curve of BLT scores segregating luminal and basal subtypes of bladder cancer. (**H**) Scatter plot of two-dimensional linear discriminant LD1 and LD2 scores in luminal, basal, and double-negative subtypes of bladder cancer. The panels in a and b were generated using the R package ComplexHeatmap (version 1.14.0). Panels C, D, E, F, and H were generated using the R package ggplot2 (version 3.2.1). Panel G was generated with pRoc (version 1.8).

We confirmed the presence of luminal and basal subtypes of bladder cancer by applying the same classification algorithm to the MDACC cohort of fresh frozen samples (n = 132)^22^. This cohort showed two major clusters of tumor samples. **(**Fig. 5A**)** The first cluster (n = 92) which included both invasive (n = 68) and non-invasive superficial bladder tumors (n = 24) was characterized by the expression of luminal markers while the second cluster, which included only invasive carcinomas (n = 35) was characterized by the expression of basal markers. Similar to the TCGA cohort, a small group (n = 5) of cases negative for both luminal and basal markers was also identified. The power of prediction of luminal and basal subtypes in the MDACC cohort was 87.8%. The prediction of double-negative subtype as compared to luminal or basal tumors was 69.5% and 85.7% respectively. Similar to the TCGA cohort, the majority of basal and luminal cases were predicted with >80%. **(**Fig. 5B–D**)** The prediction power of individual double-negative cases was lower and most of them were predicted with <80% confidence. Similar to the TCGA cohort the basal cluster contained a subset of cases with coexpression of basal and luminal markers. The BLT scores in the MDACC cohort were similar to those computed for the TCGA cohort. **(**Fig. 5E,F**)** The ROC analysis showed AUC of 0.948 (CI = 0.912–0.984) with 83.7% sensitivity and 91.4% specificity for luminal and basal subtypes at the optimal BLT cutoff point. **(**Fig. 5G**)** The two-dimensional LDA model showed 86.3% accuracy to identify luminal, basal, and double-negative subtypes. **(**Fig. 5H**)** The analyses of the EMT status confirmed the results from the TCGA cohort and showed that double-negative tumors had activated EMT with negative EMT scores. **(**Supplementary Fig. 1A–C**)** They were characterized by the upregulation of transcription factors involved in the activation of EMT and showed the downregulation of the genes encoding for homotypic adhesion proteins. **(**Supplementary Fig. 1D**)** The basal and double-negative tumors were characterized by increased immune signature revealed by immune score and CIBERSORT analyses. **(**Supplementary Fig. 2A–D**)** Similarly basal and double-negative tumors show the increased expression of genes encoding for immune checkpoints and their respective ligands including PD-L1 **(**Supplementary Fig. 2E–G**)**.

**Figure 6:**
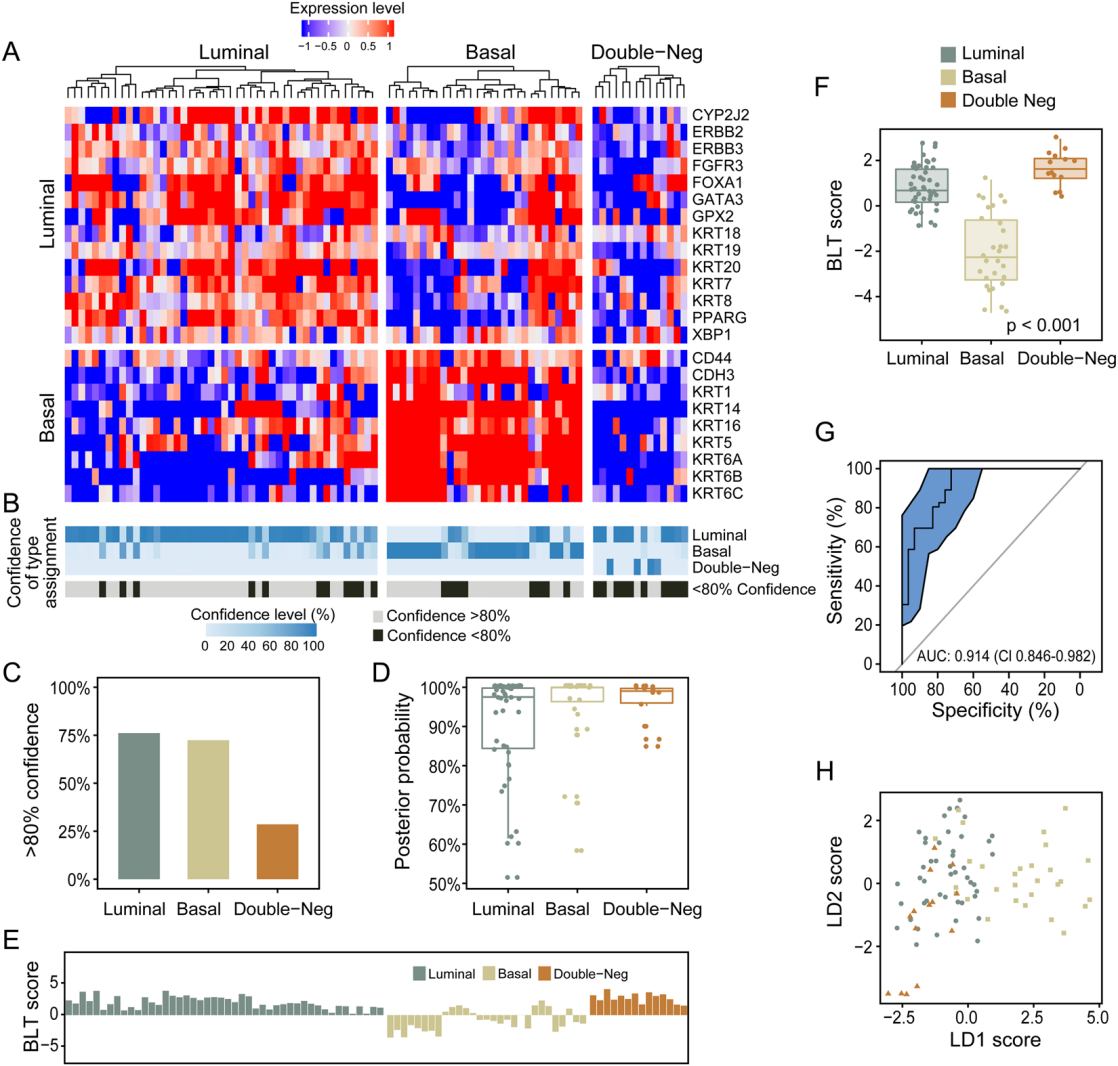
Whole-transcriptome mRNA expression profiling of the MDACC FFPE bladder cancer cohort (n = 89). (**A**) Hierarchical clustering with luminal and basal markers. (**B**) Prediction strengths of molecular subtypes. (**C**) Proportion of cases with >80% prediction strength of the molecular subtypes. (**D**) Box plot analysis of prediction strength for individual cases by posterior probability. (**E**) BLT scores in molecular subtypes of bladder cancer. (**F**) Box plot analysis of BLT scores in molecular subtypes of bladder cancer. (**G**) ROC curve of BLT scores segregating luminal and basal subtypes of bladder cancer. (**H**) Scatter plot of two-dimensional linear discriminant LD1 and LD2 scores in luminal, basal, and double-negative subtypes of bladder cancer. The panels in A and B were generated using the R package ComplexHeatmap (version 1.14.0). Panels C, D, E, F, and H were generated using the R package ggplot2 (version 3.2.1). Panel g was generated with pRoc (version 1.8).

In order to facilitate the potential use of molecular classification of bladder cancer in clinical practice, we tested the same molecular luminal/basal classifier in the MDACC FFPE cohort (n = 89) comprised entirely of high-grade muscle invasive (T_2_ and higher) bladder cancers^20^. Clustering using the luminal and basal markers showed that 46 tumors were luminal and 29 were basal while the remaining 14 were classified as double-negative. **(**Fig. 6A**)** The proportion of samples in double-negative category (15.5%) was higher in this cohort as compared to the TCGA (3.8%) and MDACC fresh frozen samples (4.2%). It is very likely that in the paraffin-embedded tissue the individual mRNA species disintegrate more often as compared to fresh frozen possibly accounting for the increased number of cases in this category. Similar to the previous two cohorts a small subset of tumor samples in the basal cluster coexpressed luminal markers. The strength of prediction of luminal and basal subtypes for the MDACC FFPE cohort was 93.9% and double-negative tumors as compared to luminal or basal were predicted with strengths of 74.9% and 65.8% respectively. The prediction strengths of individual cases by posterior probability was similar as compared to TCGA and MDACC fresh frozen cohorts. **(**Fig. 6B–D**)** The luminal FFPE bladder cancers were characterized by positive BLT scores and basal cancers had negative BLT scores. **(**Fig. 6E,F**)** The ROC analysis showed AUC of 0.914 (CI = 0.846–0.982) with sensitivity 80.4% and specificity of 82.8%. **(**Fig. 6G**)** The two dimensional LDA model showed 71.9% accuracy to identify luminal/basal and double-negative subtypes. **(**Fig. 6H**)** The analyses of EMT status and immune infiltration performed on the FFPE cohort were virtually similar to those from the two previous cohorts and showed the activation of EMT in double-negative tumors. **(**Supplementary Fig. 3A–D**)** They also showed an increased immune signature and overexpression of immune checkpoint genes including PD-L1 in both basal and double-negative tumors as compared to luminal subtype **(**Supplementary Fig. 4A–G**)**.

**Figure 7:**
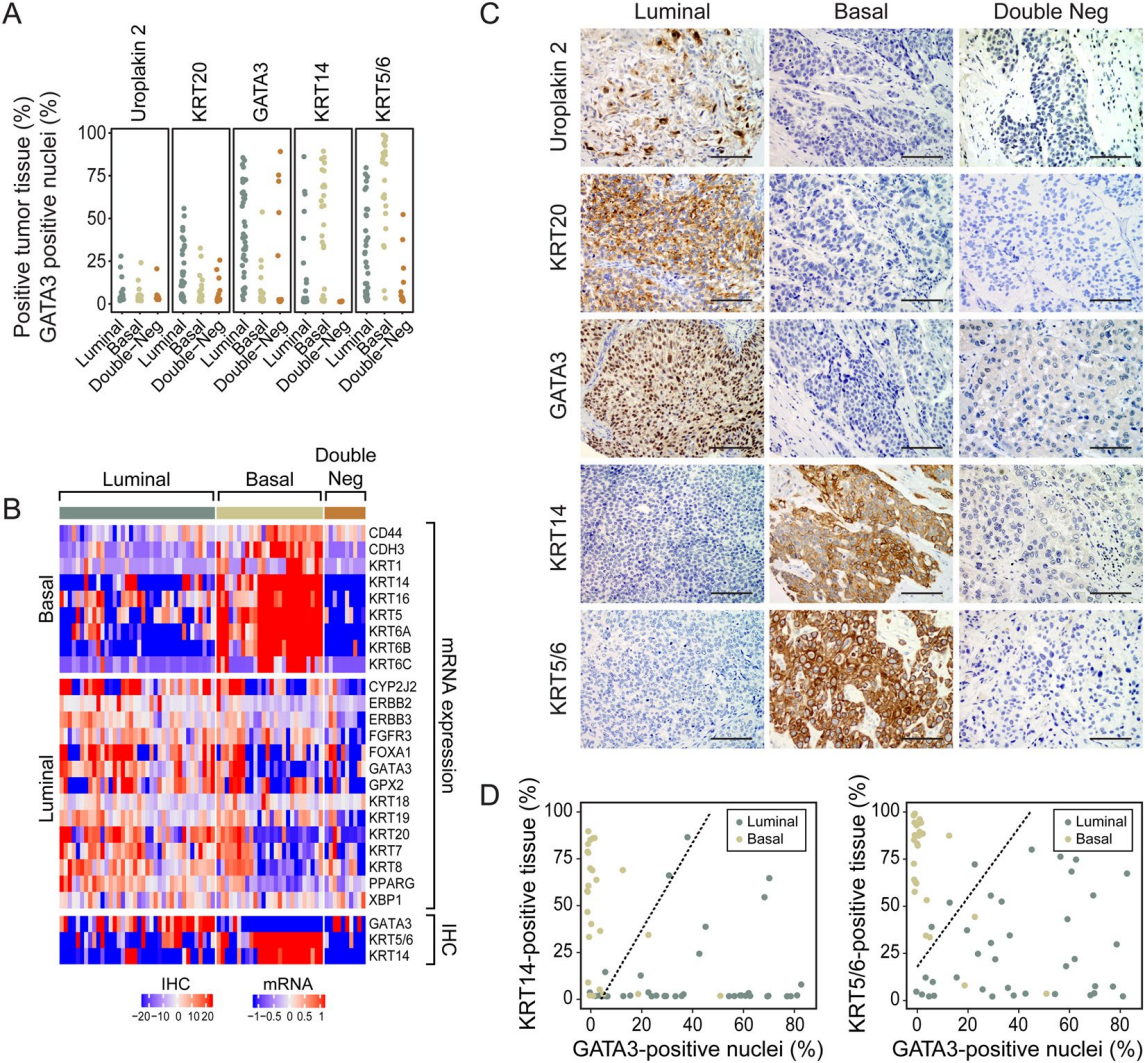
Immunohistochemical analysis of luminal and basal markers in MDACC FFPE bladder cancer cohort (n = 74) on tissue microarrays. (**A**) Quantitative image-based assessment of immunohistochemical expression levels for selected luminal (Uroplakin 2, KRT20, and GATA3) and basal (KRT14 and KRT5/6) markers. (**B**) Hierarchical clustering of luminal and basal markers using mRNA expression levels and immunohistochemically (IHC) detected levels of selected luminal (GATA3) and basal (KRT5/6 and KRT14) markers. (**C**) Examples of immunohistochemical staining patterns for selected luminal and basal markers in molecular subtype of bladder cancer. Solid bars indicate 50 µm. (**D**) Logistic regression (LRA) analyses of two pairs of immunohistochenical markers: GATA3/KRT14 and GATA3/ KRT5/6. Panels a and d were generated using the R package ggplot2 (version 3.2.1). Panel B was generated using the R package ComplexHeatmap (version 1.14.0).

In order to develop a potential immunohistochemical classifier of molecular subtypes of bladder cancer that can be used in routine clinical practice, we analyzed our previously studied 12 markers and selected five best performers which in addition showed strong positive correlations between mRNA and their respective encoded protein expression levels^30^. The five candidate markers were GATA3, KRT20, and uroplakin 2 (luminal) as well as KRT5/6 and KRT14 (basal) also routinely used in diagnostic workup of bladder tumor samples. Immunohistochemistry was performed on tissue microarrays (n = 74) that were constructed from the MDACC cohort of FFPE bladder cancer tissues. The immunohistochemical expression levels of luminal (GATA3, CK20, and uroplakin 2) and basal (CK5/6 and CK14) markers were assessed quantitatively by image analysis, which showed differential expression patterns in the respective molecular subtypes. **(**Fig. 7A**)** These results have shown that uroplakin 2 and KRT20 showed overlapping expression levels between the molecular subtypes. Among the five analyzed markers the quantitative image analyses showed promising results for GATA3, KRT5/6 and KRT14. When the results from image analyses for these three markers were compared with mRNA-based tumor subtype assignments, it became evident that the luminal tumors were enriched for the luminal markers while the basal tumors showed enrichment for expressions of the basal markers. **(**Fig. 7B**)** The examples of typical immunohistochemical staining patterns in luminal, basal, and double-negative subtypes are shown in Fig. 7C. Although there was some overlap in the expression levels of markers among molecular subtypes it appeared that the best separation can be provided by the analysis of GATA3 (luminal marker) and KRT5/6 or KRT14 (basal markers). In order to test this hypothesis we built the LR models and performed LOOCV analyses for the two potential immunohistochemical classifiers comprising GATA3 with KRT14 and GATA3 with KRT5/6. **(**Fig. 7D**)** The accuracy of luminal/basal prediction for GATA3 combined with KRT14 was 82.8% while the accuracy of molecular subtype prediction with GATA3 and KRT5/6 was 89.1%. This indicated that GATA3 and KRT5/6 immunohistochemical staining is the most effective classifier for prediction of luminal and basal molecular subtypes of bladder cancer.

**Figure 8:**
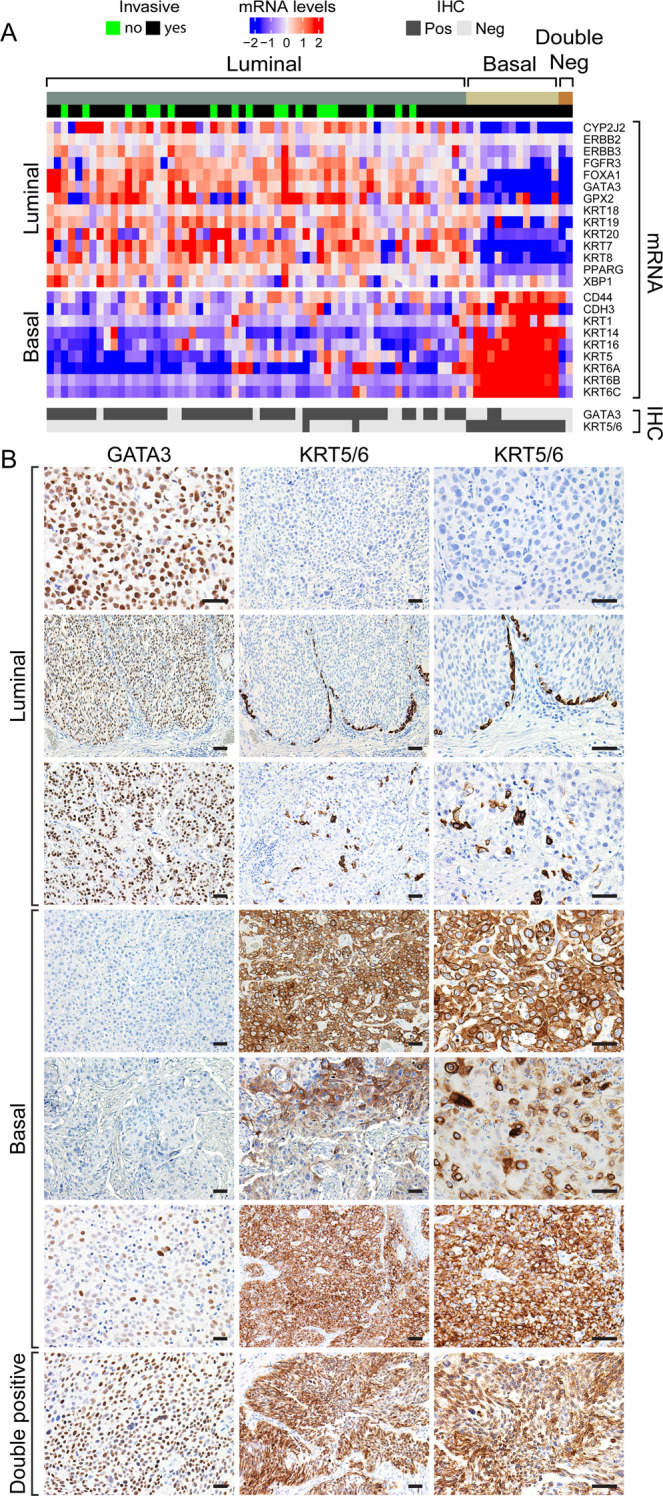
Immunohistochemical analysis of signature luminal and basal markers in different molecular subtypes of MDACC fresh frozen bladder cancer cohort (n = 74) in routine pathology sections. (**A**) Hierarchical clustering with luminal and basal markers of 74 cases from MDACC fresh frozen bladder cancer cohort in comparison to immunohistochemical expression patterns of GATA3 and KRT5/6. (**B**) Examples of immunohistochemical expression patterns of signature luminal (GATA3) and basal (KRT5/6) markers. Solid bars indicate 50 µm. Panel a was generated using the R package Complex Heatmap (version 1.14.0).

To test whether the two immunohistochemical markers (GATA3 and KRT5/6) could be used for the molecular classification of bladder cancer in daily pathology practice, we performed immunohistochemical stains for GATA3 and KRT5/6 on regular FFPE routine histologic tissue sections (n = 74) that were matched to the original MDACC cohort of fresh frozen tissue samples in which the molecular subtypes were assessed by mRNA expression levels. **(**Fig. 8A**)** The immunohistochemical staining patterns for GATA3 and KRT5/6 were analyzed in a double-blinded fashion by two experienced genitourinary pathologists (C.C.G. and B.C.) **(**Supplementary Table 5**)**. These analyses revealed that 48 (82%) out of 59 luminal tumors were positive for GATA3 only, and two (3%) showed coexpression of GATA3 and KRT5/6. The remaining nine (15%) were negative for both markers. The immunohistochemical profile of 13 basal tumors showed that 11 (85%) were positive for KRT5/6 only, and two (15%) showed coexpression of GATA3 and KRT5/6. One of the double-negative cases by mRNA expression was positive for KRT5/6 by immunohistochemistry. Review of immunohistochemically stained slides with the knowledge of their molecular subtypes provided additional insights concerning the identification of molecular subtypes of bladder cancer. **(**Fig. 8B**)** Reciprocal positivity and negativity for GATA3 and KRT5/6 was the strongest discriminatory feature between the two molecular subtypes. However, luminal tumors occasionally showed a linear layer of KRT5/6 positive cells outlining the tumor nests. In addition, they may also have scattered positive KRT5/6 cells, and this positivity was present in less than 10% of the tumor cells. In rare instances, basal tumors with strong uniform positivity for KRT5/6 showed scattered positive nuclear staining for GATA3 in less than 10% of the cells. There were also cases in which the immunohistochemical staining pattern was noninformative showing strong positivity for both markers. We concluded that the immunohistochemical staining with GATA3 and KRT5/6 is a simple classifier of molecular subtypes of bladder cancer which is effective in over 80% of the cases.

## Discussion

The current study shows that bladder cancer can be reliably classified into two molecular subtypes referred to as luminal and basal using genomic mRNA expression profiles on both fresh frozen and FFPE tumor samples. The quantitative assessment of the luminal and basal phenotypes by the BLT score identified the subtypes of bladder cancer with 80–94% sensitivity and 83–93% specificity across the cohorts. The two-dimensional LD classifier identified the luminal/basal and double-negative subtypes with 72–92% accuracy in different cohorts. The power of luminal and basal cluster assignments was in a range of 80% or above in the TCGA and MDACC cohorts. The basal tumors appeared to be heterogeneous and in all cohorts they contain a subset of cases with coexpression of basal and luminal markers. A small subset (<10%) of double-negative tumors showed unique biologic features with activated EMT state and increased immune infiltrate including overexpression of therapeutically important PD-L1. By comparing the immunohistochemical staining pattern of two markers, GATA3 and KRT5/6, with the results of mRNA-based classification, we showed that the luminal and basal molecular subtypes can be reliably identified in the majority of cases. The visual semiquantitative assessment of these markers in routine pathological preparations was shown to be a valuable tool in identifying the basic molecular subtypes of bladder cancer.

Several groups have classified bladder cancer into various molecular subtypes using genomic expression profiling^10,11,22–27^. The original genomic mRNA-based classification of bladder cancer was proposed by the Lund group which divided the disease into five subcategories^25^. TCGA group identified five distinct molecular subtypes, including luminal-papillary, luminal-infiltrated, luminal, basal/squamous, and neuronal^11^. Damrauer *et al*. and the MDACC group proposed mRNA-based classifiers dividing bladder cancer into two major groups referred to as luminal and basal^22,23^. In addition, a small fraction of bladder cancer were negative for both luminal and basal markers and were referred to as double-negative^22,23,30^. Although various groups of investigators used a different terminology for their respective subcategories, the overall set of markers is similar^10,11,22,23,27,30^. It appears that the top hierarchical level of separation is between luminal and basal categories showing features of undifferentiated basal and differentiated intermediate/luminal urothelial cell layers^18,33^.

Studies using mouse models provided evidence in support of distinct cellular origin of basal and luminal subtypes of bladder cancer. Bladder cancers developing in Upk3a-Cre^ERT2^; Trp53^L/L^; Pten^L/L^; Rosa26^LSL-Luc^ mice recapitulated the luminal molecular subtype and papillary architecture of human cancer^34^. They also showed high expression levels of Pparγ and Gata3 expression signatures consistent with their luminal origin^34^. Lineage tracing studies in the N-butyl-N-(4-hydroxybutyl)nitrosamine induced bladder cancer mouse model showed that basal cancers originate from KRT5 and sonic hedgehog-positive basal uroprogenitor cells while papillary luminal tumors are derived from the intermediate cells^35,36^.

A small fraction of tumors in our cohorts did not express neither luminal or basal markers and were referred to as double-negative. These tumors exhibitied the activation of EMT and increased immune infiltrate. In our prior studies we have shown that they were also characterized by low expression signature of claudin-related genes^30^. Recent studies confirmed this observation and showed that claudin-low bladder tumors also upregulated cytokines and chemokines with low expression levels of PPARγ allowing unopposed NF-κB activity^37^.

Molecular classification of bladder cancer provides valuable insights into its biological behavior, but using genomic mRNA expression profiling is costly and technologically complex and cannot be applied effectively in the routine clinical practice. Among the set of immunohistochemical markers, GATA3 and KRT5/6 have emerged as an effective surrogate molecular classifier of bladder cancer that correctly identified the molecular subtypes in over 80% of the cases.

In summary, we show that mRNA-based molecular classification of bladder cancer can be accomplished on both prospective fresh frozen and archived FFPE tumor samples. The luminal and basal subtypes demonstrate distinct clinicopathologic features and responses to frontline chemotherapy^18,22,28–30^. They can also be used to stratify the patients for targeted therapies including growth factors (EGFR, ERBB2, FGFR) and immune checkpoint inhibitors^14,15,38–41^. Therefore, the classification of bladder cancer in primary care centers by a simple immunohistochemical classifier has important implications for patient care.

## Methods

### Patients and study design

The genome expression profiling studies of bladder cancer were conducted on several cohorts of fresh frozen and formalin-fixed paraffin-embedded (FFPE) tumor samples obtained from MDACC’s Tissue Bank which were approved by the Institutional Review Board **(**Fig. 1 and Supplementary Table 1**)**. The tumors were classified according to the World Health Organization histologic grading system and the American Joint Committee on Cancer TNM staging system^42,43^. Two experienced genitourinary pathologists (CCG and BC) reviewed the pathological slides independently and the discrepant classifications were discussed to provide the consensus assessment. Detailed information concerning histologic grading, TNM stage, gender, race, age, smoking status for MDACC fresh frozen and FFPE cohorts are provided in Supplementary Tables 2 and 3. In addition, the TP53 mutational status is provided for the MDACC fresh frozen cohort.

The initial genomic profiling analyses were performed on fresh frozen bladder tumor samples from the TCGA (n = 408) and the MDACC (n = 132) cohorts^11,22,30^. All 408 cases in this cohort were muscle-invasive tumors (sta8888ge T2 and higher). The TCGA cohort comprised of 387 cases of high-grade and 21 low-grade carcinomas. The publicly available whole transcriptome data (RNA-seq) of tumor samples were downloaded from the TCGA website (https://tcga-data.nci.nih.gov/tcga/). Additional analyses were performed on the MDACC cohort of fresh frozen tumor samples (n = 132). The MDACC cohort comprised of 34 cases of low-grade superficial (Ta-Tis) tumors and 98 cases of high-grade invasive (T1 and higher) urothelial carcinomas. Genomic profiling studies were also performed on the MDACC cohort of FFPE tumor samples (n = 89)^20,21^. The mRNA expression data were used to formulate the quantitative basal to luminal transition (BLT) score and to develop immunohistochemical markers of bladder cancer molecular subtypes which were initially tested on the tissue microarray (TMA) (n = 74) that was parallel to the MDACC FFPE cohort. The quantitative image analysis studies of tissue microarray identified a two marker immunohistochemical classifier comprising of GATA3 and KRT5/6 which were finally validated on FFPE (n = 74) routine tissue sections that were matched to a subset of samples from the original MDACC cohort of 132 fresh frozen tumor samples.

### Genomic mRNA expression profiling

RNA from fresh frozen tissue was extracted using the mirVana miRNA isolation kit (Ambion, Inc.), and the microarray experiments were performed by direct hybridization on the Illumina HumanHT-12 v3 Expression BeadChip platform as previously described^20–22^. Data from the array images were analyzed using Illumina’s GenomeStudio.

RNA from FFPE samples was extracted using the MasterPure Complete DNA and RNA Purification Kit (Epicenter Biotechnologies, Madison, WI, USA). The microarray experiments were performed on Illumina’s WG-DASL platform as previously described^20,21^. Array data analyses were conducted with Illumina BeadStudio v3.1.3 (Gene Expression Module V3.3.8).

### Tissue microarrays and immunohistochemistry

Tissue microarrays from MDACC FFPE cohort (n = 74) were created using a tissue arrayer (Beecher Instruments, Silver Spring, MD) as previously described^8,20,21^. Based on gene expression profiles, a set of antibodies was selected for immunohistochemical analyses of luminal and basal subtypes^30^. The luminal markers included mouse monoclonal antibodies against human uroplakin 2 (BC21 clone, 1:100 dilution; Biocare Medical, Concord, CA), KRT20 (Ks20.8 clone, 1:400 dilution, Dako), and GATA3 (HG3–31 clone, 1:100 dilution; Santa Cruz Biotechnology Inc., Santa Cruz, CA). KRT14 (LL002 clone, 1:50 dilution; BioGenex, Fremont, CA) and KRT5/6 (D5/16B4 clone, 1:50 dilution, Dako) antibodies were selected as candidate basal markers. Immunohistochemical stains were performed using the Bond-Max AutoStainer (Leica Biosystems, Buffalo Grove, IL) as previously described^21^. The immunohistochemical staining patterns were quantitatively assessed by image analysis using an automated digital image analyzer, GenoMx (BioGenex, San Ramon, CA). The proportions of tumor cell nuclei positive for GATA3 staining and the proportion of positive tumor tissue for all remaining markers were measured. Additional set of whole mount FFPE sections (n = 74) corresponding to a subset of samples from the original cohort of fresh tumor samples from MDACC were stained immunohistochemically for GATA3 and KRT5/6 as described above. These sections were visually inspected by two pathologists (CCG and BC) to identify the molecular subtypes of tumors.

### Analytical pipeline

The gene signal values from the array data for the MDACC cohorts of fresh and formalin-fixed paraffin-embedded samples were transformed to logarithmic scale and normalized by the sample medians. Samples were classified into luminal and basal molecular subtypes as previously described^22^. Similar clustering analyses were performed for TCGA cohort and tumors were assigned to specific subtypes by applying the set of luminal and basal markers as described previously^22^. Initially, we performed unsupervised hierarchical clustering using the Euclidean distance metric and Ward’s linkage rule. In unsupervised clustering double-negative tumors represented a subset of luminal or basal subtypes in different cohorts. Then the double-negative tumors were manually separated as a distinct group and hierarchical clustering was repeated in three subtypes i.e. luminal, basal, and double-negative.

We calculated the prediction strength of molecular subtypes as defined by R. Tibshirani *et al*.^31^. In order to calculate the prediction strength between any two of three molecular subtypes we used the formula (2.1) implemented in the R package fpc (version 2.2) described below. Specifically, we calculated the prediction strength using the $k$ means clustering after combining samples between any two groups defined as:

$$ps(k)=\mathop{\min }\limits_{1\le j\le k}\frac{1}{{n}_{kj}({n}_{kj}-1)}\sum _{i\ne i{\prime} \in {A}_{kj}}D{[C({X}_{tr},k),{X}_{te}]}_{ii{\prime} }$$

where, ${A}_{kj}$ are the indices of the observations in the test cluster $j,\,j\in (1,\,2,\ldots k)$ and ${n}_{kj}$ is the number of observations in the same cluster. In addition, $C({X}_{tr},\,k)$ denotes clustering of samples in ${X}_{tr}$ into $k$ clusters and $D{[C({X}_{tr},k),{X}_{te}]}_{ii{\prime} }=1$ if observations *j* and $i{\prime}$ of ${X}_{te}$ are assigned to the same cluster by the training set ${X}_{tr}$ centroids. Overall, this algorithm calculates the minimum of the proportion of observation pairs in a given cluster that are also assigned to the same cluster by the training set over the $k$ test clusters. In addition, we analyzed the strength predicting the molecular subtypes for individual samples by calculating the posterior probability as defined by Bayes theorem^32^. Specifically, the prediction strength of individual cases was calculated as follows:

$$(g=luminal|x)=\frac{{f}_{luminal}(x)P(g=luminal)}{{f}_{basal}(x)P(g=basal)+{f}_{luminal}(x)P(g=luminal)+{f}_{dn}(x)P(g=dn)},$$

where $P(g=k)$ is the prior probability of the group *k* estimated by the frequency of the group $k$ in the training set, ${f}_{k}(x)$ is the density function probability of the group $k$ and $x \sim {\rm{{\rm N}}}({u}_{g=k},\,{\sum }^{})$, where ${u}_{g=k}$ is the mean of the group $k$, ${\sum }^{}$ is the covariance matrix, and $k\in (basal,\,luminal,\,dn)\,{\rm{were}}$ dn stands for double-negative. As suggested by R. Tibshirani *et al*.^31^, strength ≥80% was considered as a strong subtype prediction.

For the quantitative assessment of luminal and basal phenotypes we used the expression levels of 28 luminal and 20 basal marker genes. **(**Supplementary Table 4**)** For the assessment of luminal phenotype we used the 14 luminal markers from the original classifier^22,30^. In order to increase the power of our analyses these markers were complemented by 14 PPARγ target genes previously shown to be significantly enriched in luminal cancers^22,30^. Similarly, for the assessment of basal phenotype, we used the 9 basal markers from the original classifier and complement them with additional 11 p63 target genes which were shown to be significantly enriched in basal cancers^22,30^. Linear discriminant analysis (LDA) was performed to assess the power of individual markers to identify molecular subtypes of bladder cancer^44^. The unidimensional BLT score was defined as ${\sum }^{}{W}_{i}\,\ast \,{E}_{i}$, where ${w}_{i}$ is the negative coefficient of linear discriminant (LD) and ${E}_{i}$ is the expression of marker genes. Then a least absolute shrinkage and selection operator (LASSO) analysis was used to select the best 16 luminal and 12 basal markers to combat multicollinearity^45^. **(**Supplementary Table 4**)** Specifically, LASSO applied the L1 parameter as a constrain on the sum of the absolute values of the model parameters. In the process, 28 genes with a non-zero coefficient after the regularization process were selected for the calculation of the BLT score. We used the TCGA cohort as a training set to build a LDA model with 28 selected genes and a 5-fold cross validation procedure to assess the accuracy of the prediction. Specifically, 408 samples were equally split into five groups, in each of which the proportions of molecular subtypes were kept as the same as those of the original data set. The overall accuracy for the TCGA training set was calculated as the averaged accuracy across all 5 groups. The BLT score cutoff value was used to minimize the misclassification of subtypes and was determined through a grid searching algorithm in the R package InformationValue (version 1.2.3). The cutoff values for the TCGA, MDACC fresh frozen and MDACC FFPE cohorts were −0.26, −0.81, and −1.16 respectively.

Receiver operating characteristic (ROC) analysis, implemented in a R package pROC (version 1.14), was used to evaluate the specificity and sensitivity to classify the tumors into luminal and basal subtypes^46^. In these analyses the double-negative samples were removed and the sensitivity and specificity were calculated for the optimal point, being the closest to the top-left part of the ROC curve, defined as $MIN[{(1-sensitivity)}^{2}+{(1-specificity)}^{2}$]. The accuracy of the LDA model predictions was validated in two additional independent cohorts i.e. the MDACC fresh frozen cohort (n = 132) and the MDACC FFPE cohort (n = 89). None of the cases in the validation cohorts overlapped with the cases from the TCGA cohort used as a training set. The LASSO build LDA model was also used to compute the two dimensional LDA score designated as LD1 and LD2, which assessed the power of discrimination between the three subtypes of bladder cancer i.e. luminal, basal, and double-negative. All calculations related to LDA were implemented in a R package MASS (https://cran.r-project.org/web/packages/MASS/index.html, version 7.3)^47^.

To assess the status of EMT in molecular subtypes of bladder cancer we first analyzed the expression levels of signature transcription factors involved in the activation of EMT of SNAIL, TWIST, ZEB, FOX, SOX, and KLF families complemented with the analyses of homotypic adhesion molecules such as E-cadherin (CDH1), claudin 1 (CLDN1), and tight junction protein 1 (TJP1). To quantitatively assess the level of EMT, we calculated the EMT score based on a 76-gene expression signature reported in Byers *et al*. as previously described^21,38^. For each tumor sample, the score was calculated as a weighted sum of 76 gene expression levels: $\mathop{\sum }\limits_{i=1}^{76}{w}_{i}{G}_{ij}$, where ${w}_{i}$ is the correlation coefficient between the *i*th gene expression in the signature and that of E-cadherin and ${G}_{ij}$ is the *i*th gene’s normalized expression in the *j*th tumor sample. We centered the scores by subtracting the mean across all tumor samples so that the grand mean of the score was zero.

To analyze immune gene expression signatures for molecular subtypes of bladder cancer dendrogram nodes corresponding to genes expressed in specific immune cell types were identified through DAVID functional annotation clustering and Ingenuity Systems (www.ingenuity.com) analysis. The immune expression signature was quantitatively assessed by calculating the immune scores for the expression profile of 128 genes shown in Fig. 4A and Supplementary Figs. 2A and 4A as previously described^21,48–50^. Specifically, the immune score for the *i*th sample was defined as m_i_-(1/n) $\mathop{\sum }\limits_{j=1}^{n}{m}_{i}$, where m_i_ is the median expression level across the *i*th sample’s immune expression profile and (1/n) $\mathop{\sum }\limits_{j=1}^{n}{m}_{i}$ is the grand mean of medians across all n samples. Additional analysis of immune infiltrate was performed by the CIBERSORT algorithm (http://cibersort.standford.edu/runcibersort.php). The expression profile of 547 genes using normalized mRNA levels with absolute mode and default parameters was used to assess the presence of 22 immune cell types^51^. An empirical p value was calculated using 500 permutations to test against the null hypothesis that no cell type is enriched in each sample. Then a Fisher Exact test was used to test against the null hypothesis of no association between sample types and their statistical significance.

Logistic regression (LR) models were used to identify the relationship between molecular subtypes and immunohistochemical expression levels of signature marker proteins GATA3, KRT5/6 and KRT14^52^. Leave-one-out cross validations (LOOCV) was used to assess the accuracy of immunohistochemical markers for the prediction of subtypes^53^. The statistical analyses were performed using the R package (version 3.2.3)^54^. The ComplexHeatmap (version 1.14.0), ggplot2 (version 3.2.1), and pRoc (version 1.8) softwares were used to generate the figures^46,55,56^.

All data related to MDACC cohorts used in this study are available on GEO and their accession numbers are as follows: MDACC fresh frozen cohort, GSE48075; MDACC FFPE cohort, GSE86411.

## Supplementary information

Supplementary Information 1.

Supplementary Information 2.

Supplementary Information 3.

Supplementary Information 4.

Supplementary Information 5.

Supplementary Information 6.

Supplementary Information 7.

Supplementary Information 8.

Supplementary Information 9.

Supplementary Information 10.
