# Supplementary material for: Assessment of Luminal and Basal Phenotypes in Bladder Cancer
Source: Sci Rep. 2020 Jun 16;10:9743. doi: 10.1038/s41598-020-66747-7 (PMC7298008; doi:10.1038/s41598-020-66747-7)
Supplement: Supplementary file 1 — Supplementary Information 1. [file 41598_2020_66747_MOESM1_ESM.docx]

**Assessment of Luminal and Basal Phenotypes in Bladder Cancer**

Charles C. Guo, MD^1*^, Jolanta Bondaruk, PhD^1*^, Hui Yao, PhD^2*^, Ziqiao Wang, BS^3^,

Li Zhang, PhD^4^, Sangkyou Lee, PhD^1^, June-Goo Lee, PhD^1^, David Cogdell, MS^1^,

Miao Zhang, MD^1^, Guoliang Yang, MD^1^, Vipulkumar Dadhania, MD^1^, Woonyoung Choi, PhD^5^,

Peng Wei, PhD^3^, Jianjun Gao, MD, PhD^6^, Dan Theodorescu, MD, PhD^7^, Christopher Logothetis, MD^6^, Colin Dinney, MD^8^, Marek Kimmel, PhD^9^, John N. Weinstein, MD, PhD^2^, David J. McConkey, PhD^5^, Bogdan Czerniak, MD, PhD^1^

^1^Department of Pathology, The University of Texas MD Anderson Cancer Center, Houston, TX

^2^Department of Bioinformatics and Computational Biology, The University of Texas MD Anderson Cancer Center, Houston, TX

^3^Department of Biostatistics, The University of Texas MD Anderson Cancer Center, Houston, TX

^4^Department of Environmental Health, University of Cincinnati, Cincinnati, Ohio

^5^Johns Hopkins Greenberg Bladder Cancer Institute, Johns Hopkins University, Baltimore, MD

^6^Department of Genitourinary Medical Oncology, The University of Texas MD Anderson Cancer Center, Houston, TX

^7^Samuel Oschin Comprehensive Cancer Institute, Cedars-Sinai, Los Angeles, CA

^8^Department of Urology, The University of Texas MD Anderson Cancer Center, Houston, TX

^9^Department of Statistics, Rice University, Houston, TX

*****These authors have contributed equally to this study.

Correspondence to: Bogdan Czerniak, MD, PhD, The University of Texas MD Anderson Cancer Center, 1515 Holcombe Boulevard, Houston, TX 77030 (email: [bczernia@mdanderson.org](mailto:bczernia@mdanderson.org)).

**Supplementary Figures**

**Supplementary Fig. 1.** Dysregulation of the EMT network in the MDACC fresh frozen bladder cancer cohort (n=132). **a** Expression pattern of representative genes in the EMT regulatory network. **b** EMT scores in molecular subtypes of bladder cancer. **c** Box plot of EMT scores in molecular subtypes of bladder cancer. **d** Box plot analyses of expression levels of a signature transcription factor (ZEB2) and adhesion molecules (CDH1 and CLDN1) involved in EMT. **Panel a was generated using the R package ComplexHeatmap (version 1.14.0) panels b-d were generated using the R package ggplot2 (version 3.2.1).**

**Supplementary Fig. 2. Immune signature in the MDACC fresh frozen bladder cancer cohort (n=132). a Expression pattern of immune cell infiltrate in molecular subtypes of bladder cancer. Top to bottom: B cell, T cell, CD8, MacTH1, and dendritic cell expression clusters. Boxed areas identify samples with enrichment of immune cell infiltrate. b Box plot of immune scores calculated using the expression profile shown in a in molecular subtypes of bladder cancer. c Heat map of CIBERSORT scores for 22 immune cell types in molecular subtypes of bladder cancer. d Proportion of cases with significant CIBERSORT score in molecular subtypes of bladder cancer. e Expression of immune checkpoint genes in molecular subtypes of bladder cancer. f Box plot of immune checkpoint scores calculated using the gene expression profile in e. g Box plot of mRNA PD-L1 expression levels in molecular subtypes of bladder cancer. Panels a, c, and e were generated using the R package ComplexHeatmap (version 1.14.0). Panels b, d, f, and g were generated using the R package ggplot2 (version 3.2.1).**

**Supplementary Fig. 3. Dysregulation of the EMT network in the MDACC FFPE bladder cancer cohort (n=89). a Expression pattern of representative genes in the EMT regulatory network. b EMT scores in molecular subtypes of bladder cancer. c Box plot of EMT scores in molecular subtypes of bladder cancer. d Box plot analyses of expression levels of a signature transcription factor (ZEB2) and adhesion molecules (CDH1 and CLDN1) involved in EMT. Panel a was generated using the R package ComplexHeatmap (version 1.14.0). Panels b-d were generated using the R package ggplot2 (version 3.2.1).**

**Supplementary Fig. 4. Immune signature in the MDACC FFPE bladder cancer cohort (n=89). A Expression pattern of immune cell infiltrate in molecular subtypes of bladder cancer. Top to bottom: B cell, T cell, CD8, MacTH1, and dendritic cell expression clusters. Boxed areas identify samples with enrichment of immune cell infiltrate. b Box plot of immune scores calculated using the expression profile shown in A in molecular subtypes of bladder cancer. c Heat map of CIBERSORT scores for 22 immune cell types in molecular subtypes of bladder cancer. d Proportion of cases with significant CIBERSORT score in molecular subtypes of bladder cancer. e Expression of immune checkpoint genes in molecular subtypes of bladder cancer. f Box plot of immune checkpoint scores calculated using the gene expression profile in e. g Box plot of mRNA PD-L1 expression levels in molecular subtypes of bladder cancer. Panels a, c, and e were generated using the R package ComplexHeatmap (version 1.14.0). Panels b, d, f, and g were generated using the R package ggplot2 (version 3.2.1).**
