# Supplementary material for: Assessment of Luminal and Basal Phenotypes in Bladder Cancer
Source: Sci Rep. 2020 Jun 16;10:9743. doi: 10.1038/s41598-020-66747-7 (PMC7298008; doi:10.1038/s41598-020-66747-7)
Supplement: Supplementary file 2 — Supplementary Information 2. [file 41598_2020_66747_MOESM2_ESM.pdf]

**Supplementary Table 5. Immunohistochemical Results on Routine Histologic Tissue Sections of Bladder Cancer (n=74)**

| Markers | Luminal (59) | Basal (13) | Double Negative (2) | Total (74) |
|---|---|---|---|---|
| GATA-3 positive only | 48 (82%) | 0 | 0 | 48 |
| KRT5/6 positive only | 0 | 11 (85%) | 1 (50%) | 12 |
| Double positive | 2 (3%) | 2 (15%) | 0 | 4 |
| Double negative | 9 (15%) | 0 | 1 (50%) | 10 |
