# Supplementary material for: Assessment of Luminal and Basal Phenotypes in Bladder Cancer
Source: Sci Rep. 2020 Jun 16;10:9743. doi: 10.1038/s41598-020-66747-7 (PMC7298008; doi:10.1038/s41598-020-66747-7)
Supplement: Supplementary file 3 — Supplementary Information 3. [file 41598_2020_66747_MOESM3_ESM.pdf]

Supplementary Table 2: Summary of Clinical and Pathological Data (MDACC fresh frozen cohort; n=132)

											Smoking status			p53 Mutations	
	**Sample**	**Molecular subtype**	**Gender**	**Race**	**Age**	**Grade**	**Invasiveness**	**Pathologic stage**	**Follow up (months)**	**Current status (alive w/o disease, allive with disease, dead from disease, dead from other causes), NA**	**Smoking status**	**Pack a day**	**Years of smoking**	**Nucleotides**	**Amino Acids**
1	MDA115	Luminal	male	Caucasion	65.42	High	yes	T3N0M0	60.72	alive w/o disease	No-Smoker	NA	NA		
2	MDA94	Luminal	male	Caucasion	57.52	High	yes	T2N0M0	145.05	alive w/o disease	No-Smoker	NA	NA	c.839G>C, c.853G>A	p.R280T, p.E285K
3	MDA62	Luminal	male	African American	62.74	High	yes	T1N0M0	12.10	dead from other	Active	1 pack	44		
4	MDA44*	Luminal	male	Caucasion	60.63	High	yes	T1N0M0	23.15	dead	Active	1 1/2 packs	NA	c.811G>A	p.E271K
5	MDA7	Luminal	male	Caucasion	62.79	High	yes	T1N0M0	161.80	dead	Former	48-pack-year	45		
6	MDA150*	Luminal	female	Caucasion	68.58	High	yes	T2N0M0	137.02	alive w/o disease	Former	2-1/2 packs	NA	c.473G>A	p.R158H
7	MDA32*	Luminal	male	Caucasian	69.49	Low	no	TaN0M0	0.43	alive w/o disease	No-Smoker	NA	8		
8	MDA20	Luminal	male	Caucasion	78.96	High	yes	T3N0M0	41.44	dead	Former	7 cigarettes	50		
9	MDA97*	Luminal	male	Hispanic	67.65	High	yes	T2N0M0	94.75	dead	Active	1 pack	47		
10	MDA114*	Luminal	female	Caucasion	68.90	High	yes	T3N0M0	33.34	dead	Former	2 pack	9	c.976G>A	p.E326K
11	MDA27*	Luminal	female	Caucasion	35.72	Low	no	TaN0M0	53.11	dead	Former	NA			
12	MDA110*	Luminal	male	Caucasion	81.76	High	yes	T4N1M0	69.77	dead	No-Smoker	NA	NA		
13	MDA103*	Luminal	male	Caucasion	59.04	High	yes	T4N0M0	116.39	dead from other	Active	2 packs	NA	c.589G>A	p.V197M
14	MDA50	Luminal	female	African American	70.42	High	yes	T2N1M0	51.74	dead	No		NA		
15	MDA24	Luminal	male	Caucasion	66.80	High	yes	T3N1M0	105.28	dead	Former	80 pack-year	NA	c.839G>A	p.R280K
16	MDA101*	Luminal	male	Caucasion	82.52	High	yes	T1N0M0	96.66	dead	Former	20 pack year	NA		
17	MDA2*	Luminal	male	Caucasion	74.48	High	yes	T2N1M0	37.15	dead	No-Smoker	NA	NA		
18	MDA106*	Luminal	female	Caucasian	68.04	High	yes	T1N0M0	29.70	alive w/o disease	Active	30-40 pack years	NA	c.473G>A	p.R158H
19	MDA131*	Luminal	male	Caucasian	58.76	Low	no	TaN0M0	47.34	alive w/o disease	Former	20-pack-year	4		
20	MDA36*	Luminal	male	African American	54.01	Low	yes	T1N0M0	125.54	dead	Former	NA	NA		
21	MDA6	Luminal	female	Caucasian	47.57	Low	no	TaN0M0	146.16	alive w/o disease	No-Smoker	NA	45		
22	MDA139	Luminal	male	Caucasian	69.07	High	yes	T1N0M0	2.23	alive w/o disease	Active	1 and half pack	30		
23	MDA105	Luminal	male	African American	70.36	Low	no	TaN0M0	9.93	dead	Former	1-half pack	NA		
24	MDA82	Luminal	female	Caucasian	69.11	Low	no	TaN0M0	142.36	alive w/o disease	Former	NA	13		
25	MDA132	Luminal	male	Caucasian	63.35	High	yes	T4N0M1	47.18	dead from disease	Active	1 pack	25		
26	MDA35*	Luminal	male	Caucasian	65.72	Low	no	TaN0M0	148.23	alive w/o disease	Former	1 pack	NA		
27	MDA9	Luminal	male	Caucasian	87.21	High	yes	T1N0M0	95.70	dead	No-Smoker	NA	54		
28	MDA127*	Luminal	male	Caucasian	67.83	Low	no	TaN0M0	2.89	alive w/o disease	Former	2 packs a day	NA		
29	MDA138*	Luminal	male	Caucasian	54.13	Low	no	TaN0M0	122.85	alive w/o disease	Former	30 pack years	NA		
30	MDA90*	Luminal	male	Caucasion	55.76	High	yes	T0N0M0	100.46	daed	No-Smoker	NA	NA		
31	MDA142*	Luminal	male	Hispanic	53.79	Low	no	T0N0M0	41.44	alive w/o disease	Active	1 pack	34		
32	MDA18	Luminal	male	Caucasian	68.80	High	yes	T1N0M0	58.62	dead from disease	Former	2 packs a day	NA		
33	MDA10*	Luminal	male	Hispanic	68.10	High	yes	T1N0M0	2.46	dead from other	No-Smoker	NA	4		
34	MDA137*	Luminal	male	Caucasian	64.80	High	yes	T2N1M0	1.57	dead	Former	1 pack	NA		
35	MDA146*	Luminal	female	Caucasion	69.99	High	yes	T1N0M0	50.07	dead	No-Smoker	NA	30		
36	MDA135	Luminal	male	Hispanic	62.88	High	yes	T1N0M0	106.75	alive	Former	1 pack	NA		

Supplementary Table 2: Summary of Clinical and Pathological Data (MDACC fresh frozen cohort; n=132)

	Sample	Molecular subtype	Gender	Race	Age	Grade	Invasiveness	Pathologic stage	Follow up (months)	Current status (alive w/o disease, alive with disease, dead from disease, dead from other causes), NA	Smoking status			p53 Mutations	
											Smoking status	Pack a day	Years of smoking	Nucleotides	Amino Acids
37	MDA56*	Luminal	female	Caucasian	92.40	High	yes	T1N0M0	66.62	dead	No-Smoker	NA	NA	c.455dup	p.P153Afs*28
38	MDA102*	Luminal	male	Caucasian	58.68	High	yes	T1N0M0	24.49	alive w/o disease	No-Smoker	NA	NA	c.796C>A	p.G266R
39	MDA107*	Luminal	male	Caucasian	72.97	High	no	T1N0M0	37.38	alive w/o disease	No-Smoker	NA	43		
40	MDA129*	Luminal	male	Caucasion	72.45	High	yes	T2N0M0	52.30	dead	Active	1 pack	NA	c.473G>A	p.R158H
41	MDA55*	Luminal	male	Caucasian	76.34	High	yes	T4N2M0	18.36	dead	No-Smoker	NA	NA		
42	MDA67	Luminal	female	Caucasian	86.17	High	yes	T1N0M0	44.95	dead	Active	59-pack year	NA		
43	MDA26*	Luminal	male	Caucasian	75.47	High	no	TaN1M0	125.02	alive w/o disease	No-Smoker	NA	NA		
44	MDA104*	Luminal	male	Hispanic	65.67	High	yes	T0N0M0	121.25	alive w/o disease	No-Smoker	NA	NA	c.637C>T, c.639A>G	p.R213*, p.R213=
45	MDA119	Luminal	male	Caucasion	68.99	High	yes	T4N0M0	131.80	alive w/o disease	Former	25-pack-years	NA	c.993G>C	p.Q331H
46	MDA123*	Luminal	female	Caucasian	77.78	Low	no	TaN0M0	55.31	alive w/o disease	No-Smoker	NA	40		
47	MDA19*	Luminal	male	Caucasian	65.59	Low	yes	T1N0M0	170.33	dead	Active	NA	NA		
48	MDA28*	Luminal	male	Caucasion	49.62	Low	yes	T1N0M0	16.10	dead from other	No-Smoker	NA	42		
49	MDA65	Luminal	male	African American	58.53	Low	no	T1N0M0	26.43	dead	Active	5 cigarettes to 1-1/2 packs	NA		
50	MDA78*	Luminal	male	hispanic	54.51	Low	yes	T1N0M0	93.77	alive	Active	12 pack years	NA		
51	MDA23*	Luminal	female	Caucasion	84.95	Low	no	TaN0M0	12.33	dead	No-Smoker	NA	NA		
52	MDA148*	Luminal	male	Caucasion	57.90	Low	no	TaN0M0	26.03	alive w/o disease	Active	20 pack years	22		
53	MDA145*	Luminal	male	Hispanic	57.42	High	yes	T3N0M0	38.92	alive w/o disease	Active	1 pack	23		
54	MDA21*	Luminal	male	Caucasian	67.97	Low	no	TaN0M0	118.95	alive w/o disease	Active	1 pack a day	NA		
55	MDA22	Luminal	male	Caucasian	67.97	Low	no	TaN0M0	64.30	dead from other	Active	1/2 pack to 1 pack	NA		
56	MDA130*	Luminal	female	Caucasion	42.53	High	yes	T3N0M0	191.34	alive	Active	NA	27		
57	MDA4*	Luminal	male	Caucasian	44.14	High	yes	T0N0M0	59.90	alive w/o disease	Former	1 pack	NA	c.398T>A	p.M133K
58	MDA87	Luminal	male	Caucasian	73.01	High	no	T1N0M0	12.03	dead from other	Former	90-pack year	30		
59	MDA75	Luminal	female	Caucasian	74.79	Low	no	TaN0M0	34.07	dead	Active	1 pack	NA		
60	MDA86	Luminal	male	Caucasian	80.00	Low	no	NA	NA	NA	NA	NA	NA		
61	MDA79*	Luminal	male	Caucasian	40.95	Low	no	TaN0M0	25.84	alive w/o disease	No-Smoker	NA	40		
62	MDA1*	Luminal	male	Caucasian	69.15	Low	no	TaN0M1	99.70	alive w/o disease	Active	1 pack	29		
63	MDA52	Luminal	male	Caucasian	56.02	High	yes	T1N0M0	50.43	dead	Former	1 pack	57		
64	MDA37*	Luminal	female	Caucasian	75.90	Low	no	TaN0M1	32.43	dead	Active	1 pack	NA	c.108G>A, c.215C>G	p.P36=, p.P72R
65	MDA64*	Luminal	female	Hispanic	57.74	High	yes	T1N0M0	15.77	dead from disease	No-Smoker	NA	30		
66	MDA126	Luminal	male	Caucasian	51.30	High	yes	T0N0M0	116.13	alive w/o disease	Active	1 pack	NA		
67	MDA124*	Luminal	male	African American	53.35	High	yes	T1N0M0	39.67	alive w/o disease	No-Smoker	NA	30		
68	MDA8*	Luminal	male	Caucasian	82.89	High	yes	T2N0M0	7.54	dead from disease	Former	1/2 pack	NA		
69	MDA95*	Luminal	male	Caucasion	71.46	High	yes	T3N0M0	146.39	alive w/o disease	No-Smoker	NA	NA		
70	MDA121*	Luminal	male	Caucasian	65.65	Low	no	TaN0M0	132.16	alive w/o disease	No-Smoker	NA	42		
71	MDA122	Luminal	male	Caucasian	57.05	Low	no	TaN0M0	73.21	alive withot disease	Active	4 packs a day	30		
72	MDA71*	Luminal	female	Caucasian	67.13	High	yes	T3N1M0	12.79	dead from disease	Active	1-1/2 pack	NA		

Supplementary Table 2: Summary of Clinical and Pathological Data (MDACC fresh frozen cohort; n=132)

											Smoking status			p53 Mutations	
	Sample	Molecular subtype	Gender	Race	Age	Grade	Invasiveness	Pathologic stage	Follow up (months)	Current status (alive w/o disease, allive with disease, dead from disease, dead from other causes), NA	Smoking status	Pack a day	Years of smoking	Nucleotides	Amino Acids
73	MDA25	Luminal	male	Caucasian	83.29	Low	no	TaN0M0	114.46	dead from other	Former	23 pack-year	50	c.839G>C	p.R280T
74	MDA60	Luminal	male	Caucasian	66.35	High	yes	T3N0M0	25.21	dead	Yes	1-1/2 pack	NA		
75	MDA147	Luminal	male	Caucasian	44.94	High	no	TisN0M0	114.75	dead from disease	No-Smoker	NA	NA		
76	MDA46	Luminal	male	Caucasian	73.54	High	yes	T3N1M0	46.20	dead from disease	Active	40-pack-year	53	c.853G>A	p.E285K
77	MDA92	Luminal	male	Caucasian	73.23	High	yes	T0N0M0	21.87	dead from other	Former	1-3 packs	52		
78	MDA96	Luminal	male	Caucasian	70.70	High	yes	T1N0M0	136.36	alive w/o disease	Active	1-pack	NA		
79	MDA98	Luminal	male	Caucasian	65.26	Low	no	TaN0M0	30.66	alive	No-Smoker	NA	21		
80	MDA140	Luminal	male	Caucasian	71.95	High	yes	T3N1M0	8.69	dead from disease	Former	1 pack	NA		
81	MDA113*	Luminal	male	Caucasian	77.45	High	yes	T1N0M0	142.33	alive w/o disease	Former	15 pack-year	NA		
82	MDA89*	Luminal	male	Caucasian	78.98	High	yes	T2N0M0	18.49	alive w/o disease	Former	40-pack-year	NA		
83	MDA88*	Luminal	male	Caucasian	75.36	Low	no	TaN0M0	116.43	dead	Active	60-pack-year	NA		
84	MDA111*	Luminal	male	Caucasian	83.06	High	yes	T2N0M0	89.38	dead	Active	22 pack year	33		
85	MDA29*	Luminal	male	Caucasian	51.25	Low	no	TaN0M0	46.85	alive w/o disease	Former	2 packs	NA		
86	MDA108	Luminal	female	Caucasian	67.82	High	yes	T1N1M0	51.57	alive w/o disease	No-Smoker	NA	30		
87	MDA149	Luminal	male	Caucasian	75.68	High	yes	T3N1M0	16.85	dead	Active	2 pack	NA		
88	MDA38*	Luminal	male	Caucasion	53.66	High	yes	T3N0M0	241.11	dead	No-Smoker	NA	60		
89	MDA85*	Luminal	male	Caucasian	81.24	High	yes	T3N0M0	50.00	dead	Former	2 packs	40		
90	MDA74*	Luminal	male	Caucasian	73.67	High	yes	T3bN0M0	81.31	dead	Active	2 packs	44		
91	MDA141	Luminal	male	Caucasian	62.29	High	yes	T3N1M0	71.15	alive w/o disease	Active	30 cigarettes a day	NA		
92	MDA41	Luminal	female	Caucasian	64.85	Low	no	TaN0M0	105.64	alive w/o disease	No-Smoker	NA	40		
93	MDA47	Basal	male	African American	55.68	High	yes	T1N0M0	60.62	alive w/o disease	Former	1 pack	29		
94	MDA54*	Basal	male	Caucasian	66.96	High	yes	T2N0M1	15.77	dead from disease	Active	10 cigarettes	NA		
95	MDA120	Basal	female	African American	69.11	High	yes	T3N2M0	6.20	dead from disease	Active	12-pack-year	NA	c.403del	p.C135Afs*35
96	MDA12	Basal	male	Caucasian	40.87	High	yes	T2N0M0	25.41	dead from disease	Active	16 pack year	NA	c.743G>A	p.R249Q
97	MDA59*	Basal	male	Caucasian	62.93	High	yes	T2N2M1	15.93	alive with disease	No-Smoker	NA	10		
98	MDA51*	Basal	male	Caucasian	84.22	High	yes	T3N0M0	7.97	dead	Active	NA	40		
99	MDA134*	Basal	male	Caucasian	67.98	High	yes	T2N1M0	4.95	dead from disease	Active	1 pack	NA		
100	MDA77*	Basal	male	Caucasian	88.93	High	yes	T3N0M0	3.61	dead from other cauese	No-Smoker	NA	24	c.839G>C	p.R280T
101	MDA14	Basal	male	Caucasion	54.70	High	yes	T2N1M0	68.69	dead from other causes	Active	1 pack	NA	c.659A>G, c.853G>A	p.Y220C, p.E285K
102	MDA133*	Basal	male	Caucasian	65.38	High	yes	T0N0M0	85.05	alive w/o disease	No-Smoker	NA	50		
103	MDA70	Basal	male	Caucasian	60.11	High	yes	T3N0M0	86.46	dead	Active	1-1/2 packs	50		
104	MDA30	Basal	male	Hispanic	70.92	High	yes	T4N2M0	9.97	dead	Former	4-6 cigarettes	30		
105	MDA63*	Basal	male	Caucasian	78.66	High	yes	T2N0M1	3.41	dead from disease	Active	NA	5		
106	MDA112	Basal	male	Caucasian	52.35	High	yes	T3N2M0	60.66	dead from disease	Former	5 cigarettes	27		
107	MDA33	Basal	female	African American	73.24	High	yes	T3N0M0	14.66	dead	Active	1 pack			
108	MDA118	Basal	male	Caucasian	68.90	High	yes	T3N0M0	6.23	dead from other cause	No-Smoker	NA	41		

Supplementary Table 2: Summary of Clinical and Pathological Data (MDACC fresh frozen cohort; n=132)

											Smoking status			p53 Mutations	
	**Sample**	**Molecular subtype**	**Gender**	**Race**	**Age**	**Grade**	**Invasiveness**	**Pathologic stage**	**Follow up (months)**	**Current status (alive w/o disease, allive with disease, dead from disease, dead from other causes), NA**	**Smoking status**	**Pack a day**	**Years of smoking**	**Nucleotides**	**Amino Acids**
109	MDA11	Basal	female	African American	78.77	High	yes	T3bN0M0	20.33	dead from disease	Active	1 pack	38		
110	MDA31*	Basal	female	caucasion	63.02	High	yes	T3N0M0	59.25	alive w/o disease	Former	NA	34		
111	MDA48	Basal	male	Caucasion	72.15	High	no	T4N0M0	42.92	dead	Former	NA	4		
112	MDA128*	Basal	female	Caucasian	65.55	Low	yes	TaN0M0	18.62	dead from disease	Former	NA	NA		
113	MDA43*	Basal	male	Caucasian	65.48	High	yes	T4N0M0	127.48	alive w/o disease	No-Smoker	NA	NA		
114	MDA57*	Basal	female	Caucasion	66.39	High	yes	T4N0M1	21.31	dead from the disease	No-Smoker	NA	NA		
115	MDA49*	Basal	female	Caucasian	89.05	High	yes	T2N0M0	3.87	dead	Active	NA	30	c.108G>A, c.215C>G	p.P36=, p.P72R
116	MDA5*	Basal	male	Caucasian	76.88	High	yes	T2N0M1	1.70	dead from disease	Former	1-1/2 packs	NA	c.722C>G	p.S241C
117	MDA58*	Basal	male	African American	66.09	High	yes	T3N0M0	8.10	dead from disease	No-Smoker	NA	NA	c.991C>T	p.Q331*
118	MDA42	Basal	female	African American	69.19	High	yes	T2N0M0	128.79	alive w/o disease	No-Smoker	NA	NA		
119	MDA91*	Basal	female	Hispanic	82.83	High	yes	T3N0M0	45.08	alive with metastasis	No-Smoker	NA	NA	c.839G>C	p.R280T
120	MDA66	Basal	female	African American	80.43	High	yes	T4N1M1	4.59	dead from disease	No-Smoker	NA	NA		
121	MDA99	Basal	male	Caucasion	67.59	High	yes	T3N1M0	59.54	alive w/o disease	No-Smoker	NA	NA		
122	MDA93*	Basal	male	Caucasion	80.45	High	yes	T3N0M0	3.80	dead f/o other cause	Former	50-pack-years	25		
123	MDA53*	Basal	male	Caucasion	63.35	High	yes	T2N0M0	7.34	dead from disease	Former	2 packs	NA		
124	MDA61	Basal	male	Caucasion	68.36	High	yes	T2N1M0	32.82	dead	No-Smoker	NA	NA	c.853G>A	p.E285K
125	MDA68	Basal	female	Caucasion	58.92	High	yes	T2N1M1	6.52	dead from disease	Active	10 pack-year	NA		
126	MDA72	Basal	female	African American	77.29	High	yes	T3N0M0	109.18	dead	No-Smoker	NA	44	c.396G>C	p.K132N
127	MDA136*	Basal	male	African American	82.03	High	yes	T4N0M1	4.75	dead from disease	Former	1 pack	30	c.584T>C	p.I195T
128	MDA109*	Double neg	female	Hispanic	60.67	High	yes	T3N0M0	14.89	dead	Former	1-1/2 packs	35		
129	MDA143	Double neg	male	Caucasion	73.52	High	yes	T2N0M0	94.69	alive withput disease	Active	NA	NA		
130	MDA73	Double neg	male	Caucasian	53.79	High	yes	T2N1M0	61.54	dead	No-Smoker	NA	40		
131	MDA69	Double neg	male	Caucasian	62.67	High	yes	T3N1M0	249.08	dead	Former	NA	NA		
132	MDA34	Double neg	male	Caucasian	68.12	Low	no	TaN0M0	100.52	alive w/o disease	Former	30-pack-year	NA		

* Samples used to prepare routine pathology sections (MDACC FFPE whole-mount IHC cohort; n=74)
