# Supplementary material for: Assessment of Luminal and Basal Phenotypes in Bladder Cancer
Source: Sci Rep. 2020 Jun 16;10:9743. doi: 10.1038/s41598-020-66747-7 (PMC7298008; doi:10.1038/s41598-020-66747-7)
Supplement: Supplementary file 4 — Supplementary Information 4. [file 41598_2020_66747_MOESM4_ESM.pdf]

Supplementary Table 3: Summary of Clinical and Pathological Data (MDACC FFPE cohort; n=89)

	Luminal/Basal/Double neg	Gender	Race	Age of diagnosis	Stage	Current status (alive without disease, allive with disease, dead from disease, dead from other causes), NA	Total Follow up time after diagnosis (month)	Smoking status		
								Smoking status	Pack a day	Years of smoking
1 9464921105_D*	Luminal	male	Caucasian	61.46	T3N3M0	dead, NA	19.02	Acive	half pack	40
2 9287078069_C*	Luminal	male	Caucasian	69.05	T3N0M1	dead from disease	43.90	Former	NA	NA
3 8381670042_J	Luminal	male	Caucasian	81.33	T2N2M1	dead from disease	18.92	Former	15 cigarettes	32
4 9464921093_K	Luminal	male	Caucasian	82.95	T3N0M0	dead, NA	28.98	No-Smoker	NA	NA
5 9464921101_K*	Luminal	male	Caucasian	63.82	T3N0M1	dead from disease	13.93	No-Smoker	NA	NA
6 9287078094_A*	Luminal	female	Caucasian	62.92	T3N2M0	alive w/o disease	89.87	No-Smoker	NA	NA
7 9464921106_D*	Luminal	female	Caucasian	69.47	T3N0M0	died, other	32.92	No-Smoker	NA	NA
8 9464921093_I*	Luminal	female	Caucasian	68.84	T3N0M1	dead, NA	33.9	No-Smoker	NA	NA
9 9287078074_J*	Luminal	male	Caucasian	88.48	T3N3M0	died, other	4.98	No-Smoker	NA	NA
10 9287078069_L*	Luminal	male	Caucasian	84.07	T3N0M1	dead from disease	22.98	Former	NA	22
11 9464921101_F*	Luminal	male	Caucasian	32.29	T2N0M0	alive w/o disease	85.84	No-Smoker	NA	NA
12 9287078087_K*	Luminal	male	African-American	80.22	T3N2M1	dead from disease	16.00	No-Smoker	NA	NA
13 9287078002_K*	Luminal	male	Caucasian	82.19	T3N0M0	alive w/o disease	75.90	No-Smoker	NA	NA
14 9464921105_B*	Luminal	male	Caucasian	70.85	T4N3M0	dead, NA	10.00	Former	4-6 cigarettes	20
15 8381670042_L	Luminal	male	Caucasian	89.54	T3N2M1	dead from disease	18.92	No-Smoker	NA	NA
16 9287078021_K*	Luminal	male	Caucasian	78.39	T2N1M1	dead from disease	10.95	No-Smoker	NA	NA
17 9464921100_C	Luminal	male	Caucasian	80.01	T3N0M0	dead, NA	22.95	Active	NA	62
18 8381670042_B	Luminal	male	Caucasian	61.09	T3 N1M1	dead from disease	20.92	Former	1 pack	15
19 9464921093_G	Luminal	male	Caucasian	80.10	T3N0M0	dead, NA	26.98	No-Smoker	1 pack L20:L98	NA
20 9287078074_H*	Luminal	male	Caucasian	54.79	T3N0M1	dead from disease	21.97	Active	1 pack	35
21 9464921106_J*	Luminal	male	Caucasian	76.52	T4N2M1	dead from disease	15.93	No-Smoker	NA	NA
22 9287078002_F*	Luminal	male	Caucasian	56.44	T2N2M1	died, other	34.92	Former	2 packs	40
23 9287078074_A*	Luminal	male	Caucasian	69.18	T4N2M0	dead, NA	56.95	No-Smoker	NA	NA
24 9287078094_E*	Luminal	female	Caucasian	63.26	T3N0M0	dead, other	16.98	Former	NA	30
25 9464921106_G*	Luminal	male	Caucasian	80.86	T4N1M0	dead, NA	3.97	Former	1 pack	21
26 9464921105_L*	Luminal	male	Caucasian	69.31	T4N3M1	dead from disease	49.90	No-Smoker	NA	NA
27 9464921101_L	Luminal	male	Caucasian	85.53	T3N0M1	dead, NA	98.82	No-Smoker	NA	NA
28 9464921093_E	Luminal	male	Caucasian	60.27	T3N1M0	alive w/o disease	60.85	No-Smoker	NA	NA
29 9464921100_F*	Luminal	male	Caucasian	63.51	T2N0M1	dead from disease	12.98	No-Smoker	NA	NA
30 9287078069_A*	Luminal	female	Caucasian	81.22	T3N0M0	dead, other	80.82	Former	15 cigarettes	43
31 9287078069_D*	Luminal	male	Caucasian	70.39	T2N0M0	died, other	5.02	Active	90- pack- year	NA
32 9464921101_I*	Luminal	male	Caucasian	52.85	T3N2M1	dead from disease	12.98	Former	NA	34
33 9287078069_H*	Luminal	male	Caucasian	65.60	T3N1M0	alive w/o disease	78.92	Active	2 packs	45
34 9287078102_L*	Luminal	male	Caucasian	58.82	T3N0M1	dead from disease	34.92	Active	2 packs	38
35 9287078074_F*	Luminal	male	Caucasian	70.95	T2N0M0	NA, foreign pt	18.95	Active	2 packs	48
36 9287078002_C*	Luminal	male	Caucasian	71.52	T2N0M0	alive w/o disease	73.87	Active	NA	50
37 9464921101_A*	Luminal	male	Hispanic	65.95	T3N2M0	alive with disease	79.84	Active	80-pack-year	NA
38 9287078002_L*	Luminal	female	Caucasian	75.94	T3N0M0	dead, NA	5.02	No-Smoker	NA	NA
39 9464921101_G*	Luminal	male	Caucasian	75.41	T4N3M1	dead from disease	13.97	No-Smoker	NA	NA
40 9287078074_G*	Luminal	male	Caucasian	82.29	T4N0M1	alive with disease	8.00	No-Smoker	NA	NA
41 9464921100_B*	Luminal	female	Caucasian	75.72	T3N0M0	alive w/o disease	51.87	No-Smoker	NA	NA
42 9287078021_C*	Luminal	male	Caucasian	71.30	T3N3M1	NA, foreign pt	20.95	No-Smoker	NA	NA
43 9464921106_C*	Luminal	male	Caucasian	43.90	T3N0M0	alive w/o disease	82.92	No-Smoker	NA	NA
44 9287078021_G*	Luminal	female	Caucasian	61.04	T3N0M0	NA, foreign pt	1.93	Active	NA	1
45 9464921106_B*	Luminal	male	Caucasian	63.82	T3N0M0	dead, other	40.95	Active	70-pack-year	NA
46 9287078021_J*	Luminal	male	Caucasian	78.05	T3N0M1	dead from disease	22.92	Former	1/2 pack	10

Supplementary Table 3: Summary of Clinical and Pathological Data (MDACC FFPE cohort; n=89)

		Luminal/Basal/Double neg	Gender	Race	Age of diagnosis	Stage	Current status (alive without disease, allive with disease, dead from disease, dead from other causes), NA	Total Follow up time after diagnosis (month)	Smoking status	Smoking status	
										Pack a day	Years of smoking
47	9464921100_E*	Basal	male	Caucasian	67.30	T4N0M1	dead from disease	22.00	No-Smoker	NA	NA
48	9287078094_D*	Basal	male	Caucasian	66.59	T3N0M1	dead, probably bl ca	18.00	No-Smoker	NA	NA
49	9287078002_A*	Basal	female	Caucasian	66.33	T2N0M0	dead, NA	8.00	No-Smoker	NA	NA
50	9287078102_F*	Basal	female	Caucasian	50.46	T3N3M1	dead from disease	12.95	Active	two packs	NA
51	9287078069_K*	Basal	male	Caucasian	90.08	T3N1M1	dead from disease	23.97	No-Smoker	NA	NA
52	9287078087_J*	Basal	female	Caucasian	64.48	T4N0M1	dead from disease	34.98	Active	30-pack-year	NA
53	9287078087_D*	Basal	male	Caucasian	70.34	T3N2M0	dead from disease	13.97	Active	25 cigarettes	42
54	9464921106_L*	Basal	male	Caucasian	71.88	T3N2M0	alive w/o disease	70.92	Former	16-pack-year	NA
55	9287078074_E*	Basal	male	Caucasian	62.56	T3N0M1	dead from disease	21.97	Former	1 pack	20
56	9287078102_K*	Basal	female	African-American	76.00	T3N0M0	dead, other	3.02	No-Smoker	NA	NA
57	9464921100_D*	Basal	female	Caucasian	81.03	T3N0M0	dead from disease	14.98	No-Smoker	NA	NA
58	9464921105_G*	Basal	female	Hispanic	50.14	T3N0M0	dead, other	3.02	No-Smoker	NA	NA
59	9287078074_C*	Basal	female	African-American	73.20	T3N0M1	dead from disease	15.02	Active	1pack	27
60	9464921100_I	Basal	female	Hispanic	82.32	T3N0M0	dead from disease	22.95	No-Smoker	NA	NA
61	9287078087_B*	Basal	male	Caucasian	64.21	T3N1M1	dead from disease	8.95	No-Smoker	NA	NA
62	9287078102_E*	Basal	female	African-American	73.33	T3N0M0	dead, NA	32.00	Active	1 pack	50
63	9287078087_I*	Basal	male	Caucasian	49.54	T2N0M0	dead from disease	65.80	Active	1/2 pack	30
64	9287078021_L*	Basal	male	Caucasian	69.63	T3N3M1	dead from disease	11.97	No-Smoker	NA	NA
65	9464921106_A*	Basal	male	Caucasian	80.45	T3N0M0	dead, NA	3.02	Former	50-pack years	NA
66	9464921106_I*	Basal	male	Caucasian	82.05	T2N0M0	alive w/o disease	72.82	No-Smoker	NA	NA
67	9287078087_G*	Basal	female	Caucasian	65.30	T4N3M1	dead from disease	34.98	Former	NA	NA
68	9464921093_B*	Basal	male	Caucasian	66.08	T4N1M0	NA, no info	7.02	Former	NA	7
69	9287078021_I*	Basal	male	Caucasian	78.22	T4N0M0	dead from disease	26.89	Former	10-pack-year	NA
70	8381670042_D	Basal	male	Caucasian	73.02	T2N0M0	dead, NA	99.84	No-Smoker	NA	NA
71	9287078094_L*	Basal	female	Caucasian	83.13	T2N0M0	dead from disease	3.02	No-Smoker	NA	NA
72	9287078021_E*	Basal	male	Caucasian	76.64	T3N0M1	alive w/o disease	99.84	No-Smoker	NA	NA
73	8381670042_H*	Basal	male	Caucasian	62.21	T3N3M1	dead from disease	57.93	Active	25 packs	40
74	8381670051_B	Basal	male	African-American	44.28	T3N2M0	alive w/o disease	46.89	No-Smoker	NA	NA
75	9287078096_E*	Basal	male	Caucasian	65.70	T2N0M1	dead, sepsis	170.69	Former	2 packs	10
76	9287078094_B*	Double negative	male	Caucasian	74.71	T3N0M1	dead from disease	33.90	Former	40 packs	NA
77	9287078096_F*	Double negative	male	Caucasian	59.82	T2N1M1	dead from disease	45.87	Former	2 packs	22
78	8381670051_D	Double negative	female	Caucasian	72.27	T2N0M1	dead from disease	15.93	No-Smoker	NA	NA
79	9464921093_F	Double negative	male	Caucasian	85.41	T2N0M0	alive w/o disease	60.89	No-Smoker	NA	NA
80	9287078069_B*	Double negative	male	Caucasian	61.67	T3N0M0	alive with disease	172.62	Former	1 pack	9
81	8381670051_F	Double negative	male	Caucasian	77.39	T3N0M0	dead, NA	61.90	No-Smoker	NA	NA
82	9287078069_J*	Double negative	male	Hispanic	64.57	T3N3M1	dead from disease	23.97	Former	1 pack	16
83	9287078094_H*	Double negative	male	Caucasian	55.25	T2N1M0	alive w/o disease	122.72	No-Smoker	NA	NA
84	9287078069_G*	Double negative	male	Caucasian	70.43	T2N0M1	dead from disease	75.87	Active	1-1/2 packs	60
85	9464921101_E*	Double negative	female	Caucasian	60.72	T3N3M1	dead, NA	14.95	Former	10 pack per year	20
86	9287078102_J*	Double negative	male	Caucasian	61.25	T2N0M1	dead from disease	8.95	Former	2 packs	35
87	9464921093_J	Double negative	female	Caucasian	68.97	T2N0M1	alive w/o disease	86.85	No-Smoker	NA	NA
88	9287078102_A*	Double negative	male	Caucasian	63.76	T3N1M1	dead from disease	35.93	No-Smoker	NA	NA
89	9287078096_H*	Double negative	male	Caucasian	75.93	T3N2M1	dead from disease	23.97	Active	20 cigarettes	9

* Samples used to prepare TMA (MDACC TMA FFPE cohort; n=74)
