# Supplementary material for: Assessment of Luminal and Basal Phenotypes in Bladder Cancer
Source: Sci Rep. 2020 Jun 16;10:9743. doi: 10.1038/s41598-020-66747-7 (PMC7298008; doi:10.1038/s41598-020-66747-7)
Supplement: Supplementary file 5 — Supplementary Information 5. [file 41598_2020_66747_MOESM5_ESM.pdf]

Supplementary Table 4. List of Marker Genes Used in the Development of BLT Score

	Luminal	Basal
1	CYP2J2*	CD44 *
2	ERBB2*	CDH3*
3	ERBB3*	KRT1*
4	FGFR3*	KRT14
5	FOXA1	KRT16*
6	GATA3*	KRT5*
7	GPX2*	KRT6A*
8	KRT18	KRT6B
9	KRT19*	KRT6C*
10	KRT20*	SFN*
11	KRT7	S100A8
12	KRT8*	PI3
13	PPARG	S100A7
14	XBP1	CSTA*
15	PEPD	SERPINB2
16	ACADM	CKS2*
17	MGST1*	CCNB1*
18	CYP4B1	CCNA2
19	ACAA1	KIF23
20	CEBPA	PMAIP1*
21	ACOX1	
22	ACSL1*	
23	SCNN1G*	
24	ELOVL6*	
25	HES1*	
26	SDC1*	
27	IGFBP3	
28	GDF15*	

*Denotes markers selected for the linear discriminant analysis (LDA) by a least absolute shrinkage and selection operator (LASSO)
