# Supplementary material for: Assessment of Luminal and Basal Phenotypes in Bladder Cancer
Source: Sci Rep. 2020 Jun 16;10:9743. doi: 10.1038/s41598-020-66747-7 (PMC7298008; doi:10.1038/s41598-020-66747-7)
Supplement: Supplementary file 10 — Supplementary Information 10. [file 41598_2020_66747_MOESM10_ESM.pdf]

Supplementary Table 1. Summary of Clinical and Pathological Data for the TCGA and MDACC Cohorts.

Stage	Subtype	Gender F/M	Total	Age, yr, mean ± SD
**TCGA cohort**				
Invasive	Luminal	48/164	212	68.3 ± 11.0
(T2 and higher)	Basal	56/123	179	68.0 ± 10.1
	Double negative	3/14	17	65.6 ± 10.2
MD Anderson fresh frozen tissue cohort				
Noninvasive (Ta-Tis)	Luminal	8/24	32	64.8 ± 12.1
Invasive	Luminal	11/49	60	67.4 ± 10.6
(T1 and higher)	Basal	12/23	35	69.6 ± 10.6
	Double negative	1/4	5	63.8 ± 7.5
MD Anderson formalin-fixed paraffin-embedded tissue cohort				
Invasive	Luminal	8/38	46	70.2 ± 11.6
(T2 and higher)	Basal	11/18	29	69.2 ± 11.0
	Double Neg	3/11	14	68.0 ± 8.4

F, female; M, male; yr, year; SD, standard deviation
